# Loss of Ca^2+^/Calmodulin Dependent Protein Kinase Kinase 2 Leads to Aberrant Transferrin Phosphorylation and Trafficking: A Potential Biomarker for Alzheimer's Disease

**DOI:** 10.3389/fmolb.2018.00099

**Published:** 2018-11-20

**Authors:** Mohammad Golam Sabbir

**Affiliations:** Division of Neurodegenerative Disorders, St. Boniface Hospital Albrechtsen Research Centre, Winnipeg, MB, Canada

**Keywords:** transferrin, CaMKK2, phosphorylation, alzheimer's disease, trafficking, cerebrospinal fluid, serum, biomarker

## Abstract

Ca^2+^/calmodulin-dependent protein kinase kinase 2 (CaMKK2) is a serine/threonine kinase that is activated following an increase in the intracellular Ca^2+^ concentration and activates multiple signaling cascades that control physiologically important neuronal processes. CaMKK2 has been implicated in schizophrenia, bipolar disease, neurodegeneration, and cancer. Using isoelectric focusing (IEF) and mass spectrometry-based proteomic analysis, it was found that knockdown (KD) of CaMKK2 in cultured adult primary dorsal root ganglion (DRG) neurons resulted in the reduction of transferrin (TF) phosphorylation at multiple functionally relevant residues which corresponded to loss of an acidic fraction (pH~3-4) of TF. *In vitro* studies using CRISPR/Cas9 based CaMKK2 knockout (KO) HEK293 and HepG2 cells lines validated previous findings and revealed that loss of CaMKK2 interfered with TF trafficking and turnover. TF is an iron transporter glycoprotein. Abnormal accumulation of iron and/or deregulated Ca^2+^ homeostasis leads to neurodegeneration in Alzheimer's disease (AD). Therefore, it was hypothesized that aberrant CaMKK2 in AD may lead to aberrant phosphorylated transferrin (P-TF: pH~3-4 fraction) which may serve as a hallmark biomarker for AD. A significant reduction of P-TF in the brain and serum of CaMKK2 KO mice and a triple-transgenic mouse model of AD (3xTg-AD) supported this hypothesis. In addition, analysis of early (< 65 years) and late-stage (>65 years) postmortem human AD cerebrospinal fluid (CSF) and serum samples revealed that aberrant P-TF (pH~3-4 fraction) profile was associated with both early and late-stage AD compared to age-matched controls. This indicates P-TF (pH~3-4 fraction) profile may be useful as a minimally invasive biomarker for AD. In addition, this study provides a link between aberrant CaMKK2 with TF trafficking and turnover which provides a novel insight into the neurodegeneration process.

## Introduction

Calcium ions (Ca^2+^) are one of the most ubiquitous second messengers which play a crucial role in many signaling pathways, especially in neuronal tissues (Kawamoto et al., 2012). Calmodulin (calcium-modulated protein: CaM) is an intracellular Ca^2+^ receptor which is able to bind four Ca^2+^ ions (Swulius and Waxham, 2008). The Ca^2+^/CaM dependent protein kinase kinase 2 (CaMKK2) is a serine/threonine (Ser/Thr) kinase that becomes activated by Ca^2+^/CaM binding (Racioppi and Means, 2012). Active CaMKK2 subsequently phosphorylates and activates three major downstream kinases, CaMKI, CaMKIV, and AMP-activated protein kinase (AMPK) (Marcelo et al., 2016), which lead to the regulation of cell growth as observed in neurite elongation and branching (Wayman et al., 2004), cell cycle control (Kahl and Means, 2004), energy balance (Anderson et al., 2008, 2012; Lin et al., 2011), gene expression and protein synthesis (Oury et al., 2010; Lin et al., 2015). Disruption of Ca^2+^ homeostasis has been linked to a variety of metabolic diseases (Marcelo et al., 2016). Dysregulation of intracellular Ca^2+^ homeostasis is considered an underlying factor in the development of Alzheimer's diseases (AD) (Hermes et al., 2010; Berridge, 2011). CaMKK2 is expressed ubiquitously and has its strongest expression in the human brain (Uhlén et al., 2015). In order to understand the role, CaMKK2 was knocked down (KD) in cultured primary adult rat dorsal root ganglion (DRG) neurons and total cellular proteins were profiled based on the net electrical charge (isoelectric point: pI) and molecular weight. Protein profiling followed by mass spectrometric analysis revealed a significantly reduced acidic fraction (pI/pH~3-4) of the transferrin (TF) containing multiple phosphorylated serine (Ser), threonine (Thr) and tyrosine (Tyr) residues in the CaMKK2 KD neurons compared to the control.

TF is an iron transporter glycoprotein. Iron is an integral part of haem and iron-sulfur (Fe-S) cluster and acts as a co-factor for numerous key enzymes involved in metabolic reactions (Rouault, 2013). Free iron can promote free radical formation resulting in the oxidative damage (Gomme et al., 2005). Therefore, iron is transported safely in a redox-inactive state by the TF. Circulating TF captures iron released into the plasma mainly from the intestinal enterocytes or reticuloendothelial macrophages (Abbaspour et al., 2014) which then binds to the cell-surface TF receptor (TFR) and gets internalized (Gomme et al., 2005). The internalized iron may be donated to cytosolic target proteins through chaperons (Philpott, 2012), trafficked to the mitochondria for the synthesis of haem or Fe-S clusters (Barupala et al., 2016), or stored in cytosolic ferritin (Arosio et al., 2009). Dysregulation of iron metabolism contributes to various human pathologies including iron overload diseases (Fleming and Ponka, 2012), neurodegenerative brain diseases (Rouault, 2013), atherosclerosis (Sullivan, 1981) and cancer (Bogdan et al., 2016). Interestingly, in a genome-wide analysis of the human kinases involved in endocytosis revealed that silencing of the CaMKK2 in HeLa cells leads to decreased accumulation of fluorescent-TF in enlarged cytoplasmic structures which indicates defective TF trafficking or signaling (Pelkmans et al., 2005). Therefore, it was hypothesized that loss of CaMKK2 mediated reduction of TF phosphorylation may be associated with TF trafficking and turnover.

The Ser/Thr kinase, cyclin-dependent kinase 5 (CDK5) and glycogen synthase kinase 3 (GSK3) have been identified as upstream kinases that increase the stability and autonomous activity of the CaMKK2 (Green et al., 2011). GSK3 has been implicated in AD (Hooper et al., 2008; Kremer et al., 2011; Llorens-Martin et al., 2014) and Parkinson's disease (PD) (Morales-Garcia et al., 2013; Golpich et al., 2015; Choi and Koh, 2018). CDK5 is a neuron-specific kinase that has been linked to an array of neurodegenerative disorders including AD, PD and Huntington's disease (HD) (Cheung and Ip, 2012; Kawauchi, 2014). In addition, a decline in cAMP-dependent protein kinase A (PKA) signaling, another CaMKK2 upstream kinase (Wayman et al., 1997; Cao et al., 2011), contributes to the etiology of several neurodegenerative diseases, including AD and PD (Dagda and Das Banerjee, 2015). The iron level generally increases in the aging brain (Bartzokis et al., 1997) but in AD and PD, brain iron content shows a dramatic increase (Altamura and Muckenthaler, 2009). In AD, iron accumulates in the same brain regions that are characterized by the hallmark amyloid-β peptide (Aβ) deposition, such as the hippocampus, parietal cortex and motor cortex (Dedman et al., 1992; Good et al., 1992). CaMKK2 downstream effector AMPK was found deregulated in the brain of AD patients where it co-localized with phosphorylated tau protein in pre-tangle and neurofibrillary tangle (NFT)-bearing neurons (Vingtdeux et al., 2011). NFTs are a pathological hallmark of neurodegenerative diseases that contains intraneuronal aggregates of hyperphosphorylated and misfolded tau (Serrano-Pozo et al., 2011). A recent study has shown that AMPK modulated tau phosphorylation and tau pathology *in vivo* (Domise et al., 2016). In PD, one of the pathological hallmarks is neurodegeneration with brain iron accumulation and diffuse Lewy body formation (Altamura and Muckenthaler, 2009). The Lewy bodies are mainly composed of α-synuclein protein aggregates (Goedert, 2001). Multiple studies have now shown that iron promotes aggregation of the α-synuclein (Hashimoto et al., 1999; Golts et al., 2002). While the mechanism for the brain iron accumulation in these disorders is unknown, it correlates with the production of ROS and oxidative damage that hallmark these neurodegenerative disorders (Altamura and Muckenthaler, 2009). Therefore, it was further hypothesized that aberrant CaMKK2 may lead to the aberrantly phosphorylated TF during the development and progression of AD. In addition, the aberrant P-TF (pH~3-4 fraction) profile will be reflected in the serum or cerebrospinal fluid (CSF) of AD patients and therefore, can be used as a potential biomarker for AD.

The aim of the present study was to validate loss of CaMKK2 mediated aberrant TF phosphorylation and trafficking using CaMKK2 KO mice and multiple CRISPR/cas9 based CaMKK2 KO human cell lines. An additional aim was to identify and validate P-TF as a potential biomarker for AD using triple-transgenic AD (3x-Tg-AD) mouse model as well as CSF and serum samples from postmortem early and late-onset AD (EOAD: < 65 years and LOAD:>65 years) patients. This study reports that CaMKK2 controls TF phosphorylation, intracellular trafficking, and turnover *in vivo*. In addition, it was shown that P-TF (pH~3-4) profile may serve as a novel serum or CSF based biomarker for AD.

## Materials and methods

### Transgenic mouse (CaMKK2 KO and 3xTg-AD) and postmortem human extracellular fluids from alzheimer's patients

The CaMKK2 KO male mouse brain and spinal cords were provided as dissected snap-frozen tissue by Dr. Uma Sankar, Indiana University School of Medicine, USA. The CaMKK2 KO mouse was generated by targeted deletion of exons 2–4 flanking sequence (Anderson et al., 2008). The CaMKK2 KO mice used in this study were from C57BL/6J background and approximately 3–4 months old. The 6 and 14 months old female 3xTg-AD mice cerebral cortex, hippocampus and serum samples were provided by Dr. Benedict C Albensi, University of Manitoba, Canada. The 3xTg-AD is a triple-transgenic model of AD harboring PS1(M146V), APP(Swe) and tau (P301L) transgenes (Oddo et al., 2003). The postmortem human CSF and serum samples from AD patients and unaffected controls were obtained through National Institute of Health (NIH) NeuroBioBank, USA (Request number #937).

### Cell culture

DRGs from adult male Sprague-Dawley rats were dissected and dissociated as described previously (Calcutt et al., 2017; Sabbir et al., 2018) and cultured in defined Hams F12 media containing 10mM D-glucose (N4888, Sigma, St Louis, MO, USA) supplemented with modified Bottenstein's N2 additives without insulin (0.1 mg/ml TF, 20 nM progesterone, 100 μM putrescine, 30 nM sodium selenite, 0.1 mg/ml BSA; all additives were from Sigma, St Louis, MO, USA) (Akude et al., 2011; Roy Chowdhury et al., 2012; Saleh et al., 2013; Calcutt et al., 2017). In all experiments, the media was also supplemented with 0.146 g/L L-glutamine, a low dose cocktail of neurotrophic factors (0.1 ng/ml NGF, 1.0 ng/ml GDNF and 1 ng/ml NT-3 – all from Promega, Madison, WI, USA), 0.1 nM insulin and 1X antibiotic antimycotic solution (A5955, Sigma).

### Generation of CaMKK2 KO cell lines

The HEK293 cell line was a gift from the Dr. Asuka Inoue laboratory, University of Tohoku, Japan. The HepG2 cell line was a gift from Dr. Jeffrey Wigle laboratory, University of Manitoba, Canada. The cells were cultured in DMEM supplemented with heat-inactivated 5% FBS, 0.146 g/L L-glutamine and 1X antibiotic antimycotic solution (A5955, Sigma). The CaMKK2 KO cell lines were generated by transfecting HEK293 and HepG2 cells with CaMKK2 CRISPR/Cas9 KO plasmid (Sc-400928) and CaMKK2 homology-directed repair (HDR) plasmid (Sc-400928-HDR) using lipofectamine reagent (Table 1). The cells were selected with 2 μg/ml puromycin following 24 h of transfection and subsequently plated as single cell/well in 96 well plates and allowed to develop colonies which were then screened for the reporter red fluorescent protein (RFP) and CaMKK2 protein expression by confocal microscopic imaging and immunoblotting, respectively. The floxed-RFP reporter cassette in the HDR construct was excised by transiently expressed by Cre-recombinase vector (Sc-418923). The lists of CRISPR/Cas9 constructs are given in Table 1.

**Table 1:** List of Antibodies and other reagents.

**Name**	**Description**			**Source**	**Catalog number**
CaMKK2 HDR plasmid (Human)	Homology arm with selection/reporter cassette for HDR			SCBT	Sc-400928-HDR
CaMKK2 CRISPR/Cas9 KO plasmid (Human)	Pool of 3 plasmids each encoding ccas9 nuclease and a target specific 20nucleotide gRNA			SCBT	Sc-400928
Cre vector	Cre recombinase expressing plasmid			SCBT	Sc-418923
mCherry-TFR	C-terminal mCherry tagged TFR			Addgene	55144
Flag-TF cDNA	Human Transferrin full length, N-terminal Flag tag			Gene Script	OHu26129
CaMKK2 siRNAs: s135956, s135958, s135957	Rat CaMKK2 specific siRNAs: CGAGCAGGTGTATCAGGAA, CAAAGGCATTGAGTACTTA, CCCGGATGTTGGACAAGA			ThermoFisher Scientific	4390771
**Name**	**Source**	**Type**	**Host Sp**.	**Cat. No**	**Lot No**.
**Antibodies**					
CaMKK2(ZZ9)	SCBT	Monoclonal	Mouse	Sc-100364	I2914
B-23(NA24)	SCBT	Monoclonal	Mouse	Sc53175	F2212
VDAC1(B-6)	SCBT	Monoclonal	Mouse	Sc-390996	C0116
α-tubulin (TU-02)	SCBT	Monoclonal	Mouse	Sc-8035	C2017
ERK1/2(C-9)	SCBT	Monoclonal	Mouse	Sc-514302	G3115
Histone-1(G-1)	SCBT	Monoclonal	Mouse	Sc-395530	G1514
GAPDH(0411)	SCBT	Monoclonal	Mouse	Sc-47724	1015
Flag	Sigma	Monoclonal	Mouse	F3165	SLBT6752
Phospho-Serine	SCBT	Monoclonal	Mouse	SC-81514	I1615

### Knockdown of CaMKK2

Knockdown of CaMKK2 in cultured DRG neurons was achieved by transfecting cells with lipid nanoparticles (LNP) encapsulated cocktail of 3 siRNAs specific to exon 5, 8, and 12 of CaMKK2 gene (Table 1) (Rungta et al., 2013). The siRNA-LNPs were prepared by mixing appropriate volumes of different cationic lipid stock solutions in ethanol with an aqueous phase containing siRNA multiplexes using a microfluidic micromixer by Precision NanoSystems Inc. For encapsulation, the desired amount of siRNAs (0.056 mg siRNA/ micromole of lipid) was dissolved in the formulation buffer (25 mmol/L sodium acetate, pH 4.0). Subsequently, 1 × volume of the lipid mixtures in ethanol and 3 × volumes of the siRNA in formulation buffer were combined in the microfluidic micromixer using a dual syringe pump to generate the LNPs. The LNP particles containing siRNA were added to the DRG culture and neurons were allowed to grow for 48 h, after which the proteins were analyzed. The LNP based delivery of siRNA was not cytotoxic and did not affect the viability of DRG neurons after 48–74 h of exposure as reported previously in the neuronal cells (Rungta et al., 2013).

### Two-dimensional (2D) IEF followed by SDS-polyacrylamide gel electrophoresis (IEF/SDS-PAGE)

IEF separates proteins based on the isoelectric point (pI) which depends on the net charge in the protein. During focusing, the proteins will migrate to the point on an immobilized pH gradient where the net charge of the protein is zero (Freeman and Hemby, 2004). The charge-separated proteins were further separated in the 2nd dimension SDS-PAGE based on their molecular weight. Total cellular proteins were precipitated and dissolved in the rehydration buffer containing 8 M Urea, 2% CHAPS, 50 mM dithiothreitol (DTT) and 0.2% Bio-Lyte ampholytes pH3-10 (Bio-Rad, Cat no. 1632094). The dissolved proteins were then incubated in IPG strips (ThermoFisher) for 1 h and focused at 175 volts (V) for 15 min, 175–2,000 V ramp for 45/109 min (depending on pH gradient) and 2,000 V for 30 min. In some experiments, IPG strips from GE Healthcare Life Sciences Immobiline DryStrip (pH 3–10 L) and Bio-Rad ReadyStrip IPG strips (pH 4-7L) were used and the proteins were focused using Bio-Rad Protean®i12 IEF system as per manufacturer's recommendations. After focusing, the proteins in the strips were reduced (by DTT), alkylated (by Iodoacetamide) and resolved by 2D SDS-PAGE. For protein profiling experiments, the gel was stained with colloidal Coomassie, imaged and the protein spots were compared (Dyballa and Metzger, 2009). For immunoblotting experiments, the gels were transferred to nitrocellulose membrane and immunoblotted using different antibodies (Table 1).

### In-gel digestion and mass spectrometry

In-Gel digestions of the excised gel-spots were performed as follows. The spots were sliced, destained, dehydrated and dried. the dried gel slices were then rehydrated in 20 μl of 12 ng/μl Trypsin Gold (V5280, Promega) in 0.01% ProteaseMAX^TM^ Surfactant (Trypsin enhancer, V2071, Promega):50 mM NH_4_HCO_3_ for 10 min and then overlaid with 30 μl of 0.01% ProteaseMAX^TM^ Surfactant:50mM NH_4_HCO_3_, gently mixed and incubated overnight at 37°C on a horizontal shaker. The eluted peptides were cleaned by Pierce^TM^ C-18 tips (ThermoFisher, 87782) and analyzed by tandem mass spectrometry (MS) analysis using AB SCIEX TripleTOF™ 5600 System (Applied Biosystems/MDS Sciex, Foster City, CA) at Manitoba Centre for Proteomics and Systems Biology, University of Manitoba.

### Formaldehyde cross-linking and immunoprecipitation

The HEK293 cells were transiently transfected with Flag-TF in serum-starved condition for 14 h. The cells were then crosslinked using 2% freshly prepared paraformaldehyde in phosphate-buffered Saline (PBS) for 10 min. The crosslinking was stopped with Tris and washed thrice in PBS for 10 min each. The protein lysates were prepared in PBS containing 1% IGPAL630 (Sigma), sonicated and reduced (DTT). The crosslinked samples were not boiled but the native proteins were boiled for 10 min in a water bath. The Flag-TF was immunoprecipitated by incubating the protein lysates with 8 μg anti-Flag antibody/50 ul Dynabeads protein-G (F3165: Sigma, 10003D: ThermoFisher Scientific) for overnight and washed thrice using PBS with 1% IGPAL630 for 10 min each. The proteins were denatured, reduced and separated by SDS-PAGE. The protein gels were stained with Oriole fluorescent stain (Bio-Rad, Cat no.1610496) or transferred to nitrocellulose membrane and immunoblotted.

### Two-dimensional blue native polyacrylamide gel electrophoresis followed by SDS-PAGE (BN-PAGE/SDS-PAGE)

In BN-PAGE/SDS-PAGE, the first dimension native page separates multiprotein complexes and the 2nd dimension denatured and reduced SDS-PAGE separates the interacting protein components in the MPC which appears on a vertical line (Sabbir et al., 2016). The first dimension BN-PAGE and the 2nd dimension SDS-PAGE was performed as described previously (Sabbir et al., 2016). Briefly, the cell lysates were prepared in 1X PBS supplemented with 1X Halt protease and phosphatase inhibitor cocktail (1861281, Thermo Scientific) and 1.5% *n*-Dodecyl β-D-maltoside (D4641, Sigma) and sonicated. The proteins were then separated in 4–15% gradient blue-native polyacrylamide gel. The gel strips (individual lanes) were carefully excised including the 3.2% stacking gel and immersed in the Laemmli sample buffer containing freshly prepared DTT (54 mg/ml). The gel slices were incubated in sample buffer for 30 min at 55°C temperature and then the proteins in the gel slices were separated in the 2nd dimension SDS-PAGE and immunoblotted.

### Transferrin uptake assay

The FITC-conjugated TF uptake assay was performed by overexpressing RFP tagged TFR plasmid (Table 1) in wild-type and CaMKK2 KO HEK293 cells. The cells were grown overnight in serum-free media and then incubated with 25 μM FITC-TF in serum-free media. Live confocal time-lapse images were taken using an environment controlled LSM510 confocal microscope. The cells were also pulse labeled with 25 μM FITC-TF for 1 h and then washed and overnight incubated in serum-free media and imaged. LSM510 confocal images acquisition parameters were the same for all images.

### Calcium response assay

Wild-type and CaMKK2 KO (clone A5) cells were cultured for 48 h in serum-free media. The culture media was then replaced with a salt-glucose solution containing 114 mM NaCl, 0.22% NaHCO3, 5.29 mM KCl, with or without CaCl2.2H2O (with calcium: 2 mM CaCl2+1 mM BaCl2; without calcium: 3 mM BaCl2), 10 mM HEPES, 10 mM Glucose, 1 mM MgCl2 and supplemented with 5 μM Fluo-4AM dye (ThermoFisher, F14210). The cells were loaded with cell-permeant Fluo-4 for 15 min, washed and then time series images were captured at 30 s interval following 10 μM Muscarine (Sigma, M6532) treatment. Zeiss LSM410 confocal microscope with a controlled humidified atmosphere containing 5% CO2 at 37°C was used to capture time lapse images. The mean Fluo-4 intensity was calculated from time series images using ImageJ time-series analyzer plugin.

### Western blotting and chemiluminescence-detection

Relative quantification of proteins was done by SDS-PAGE separation of total proteins followed by transfer to the nitrocellulose membrane and immunoblotting based detection using HRP-conjugated secondary antibodies. The chemiluminescence was detected and imaged using ChemiDoc^TM^ imaging system and Image Lab software version 5.0 build-18 (BioRad). Table 1 summarizes all the primary antibodies and other reagents used in this study.

### Statistical analysis

Statistical analysis was performed using Prism version 7.00 (GraphPad Software). The mean of more than 2 groups were compared using one-way ANOVA (randomized) followed by multiple comparison tests (Siegel, 1956; Dunn, 1964). The mean of multiple experimental groups were compared with the control group by Dunnett's *post-hoc* multiple comparison test, whereas, the mean between two experimental groups were compared by Sidak's *post-hoc* multiple comparison test (Dunn, 1964). Comparisons between two groups were performed using Student's *t*-test (unpaired). Differences were considered significant with *P* <0.05.

## Results

### CaMKK2 KD in DRG neurons leads to loss of negatively charged fraction (pH~3-4) of TF containing multiple phosphorylated residues

**Figure 1:**
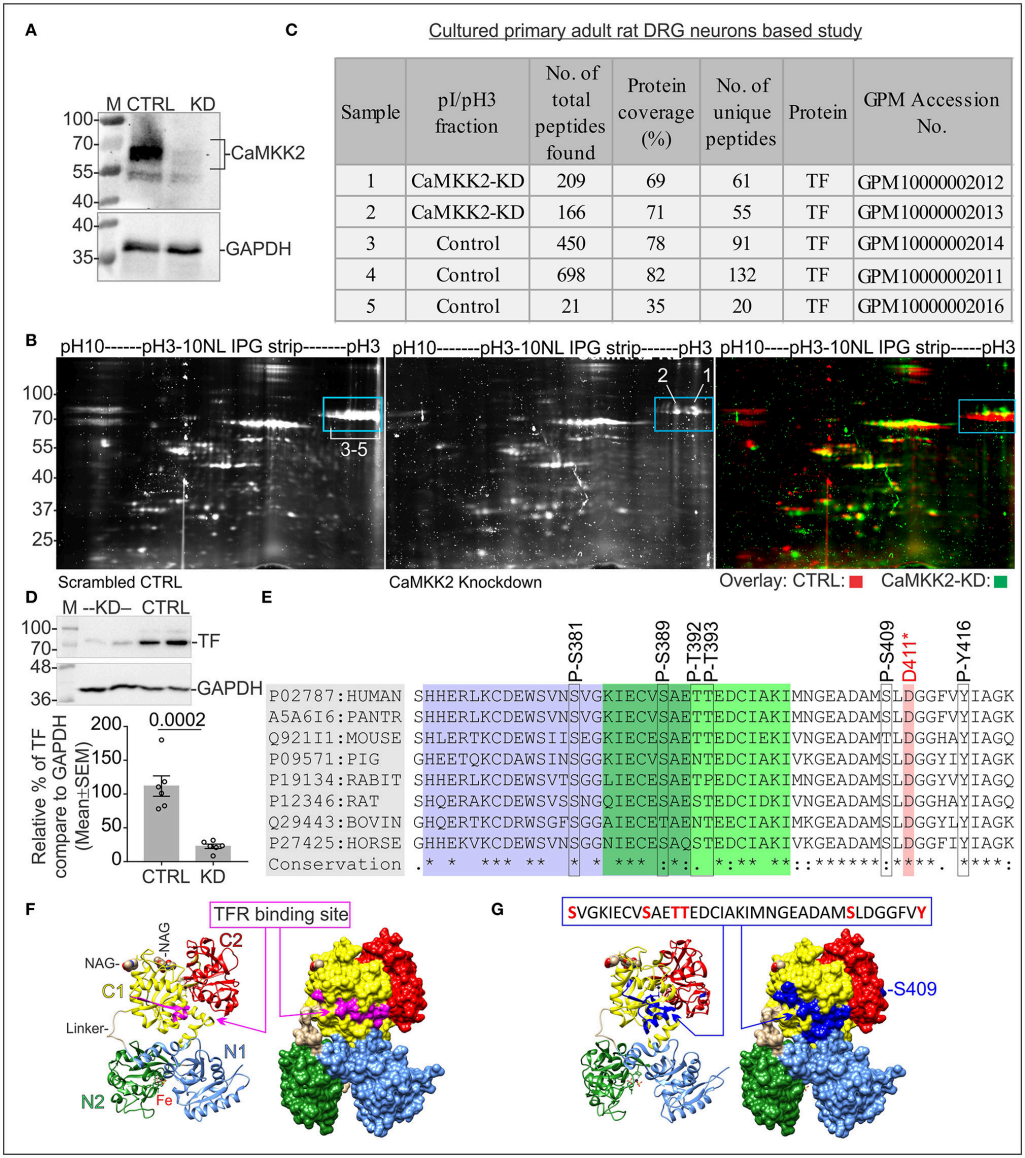
Protein profiling in CaMKK2 KD cultured adult primary rat DRG neurons. **(A)** Immunoblots showing expression of CaMKK2 and GAPDH. CTRL, scrambled control, KD, knockdown. The LNP based delivery of siRNAs knocked down 98% of CaMKK2 in DRG neurons. The protein lysates were obtained from DRG neurons cultured for 48 h following transfection. **(B)** Oriole stained IEF/SDS-PAGE gel showing focused proteins. Blue rectangle areas show the difference in the focused protein fractions. The gel images (CTRL and KD) were false-colored and overlaid in the right panel to highlight the differences. Arabic numerals indicate gel spots that were used for in-gel trypsin digestion and mass spectrometric identification of the proteins. The numbers are equivalent to the sample numbers in **(C)**. **(C)** Table summarizes mass spectrometry findings. Data is accessible at Global Proteome Machine (https://thegpm.org) website using the GPM identifiers. **(D)** Immunoblots showing expression of TF and GAPDH. CTRL, scrambled control; KD, knockdown. The protein lysates were obtained from DRG neurons cultured for 72 h following transfection. Bottom panel: representative scatter plot showing the relative abundance of TF normalized to GAPDH expression. *N* = 6 replicates from 3 independent experiments. The *p*-value by *t*-test (unpaired). **(E)** Conserved TFR binding motif in TF in different mammalian species showing the P-TF residues identified by mass spectrometry. D-411 residue is involved in iron binding. The purple and green highlighted residues indicate the TFR binding site identified by cryo-EM (Cheng et al., 2004) and epitope mapping (Teh et al., 2005). **(F)** Ribbon representation of hTF showing TFR binding site (Wally et al., 2006). TF modeled using diferric bound human serum TF crystal structure, PDB: 3QYT. The subdomains: N1 (residues 1-92 and 247-330) in blue, N2 (Residues 93-246) in green, C1 (Residues 340-325 and 573-679) in yellow, C2 (residues 426-572) in red, and the peptide linker in brown. Fe(III) ions are represented as sphere models in black. The two N-acetyl-glucosamine moieties (NAG and NAG') are represented as sphere models. Molecular graphics were prepared using UCSF Chimera package (Pettersen et al., 2004). Right panel: molecular surface presentations showing TFR binding site highlighted in pink. **(G)** Ribbon and molecular surface model of TF showing epitopes containing the phosphorylated residues (blue).

In order to understand the role of CaMKK2 in neurons, CaMKK2 was knocked down in cultured adult rat primary DRG neurons (Figure 1A) and total cellular proteins were fractionated by IEF/SDS-PAGE to detect and compare differentially charged protein fractions between control and KD samples (Figure 1B). Comparison of the focused proteins revealed the difference in the abundance of multiple ~75 kDa protein spots at pH~3-4 (Figure 1B, blue rectangles), excised and analyzed by in-gel tryptic digestion followed by mass spectrometric identification of the proteins (Figure 1C). Mass spectrometry identified the protein spots as TF and revealed multiple potential phosphorylated Ser-381/389/409/500/511/512, Thr-392/393/586, and Tyr-257/333/336/338 residues in the scrambled control sample which was absent in KD condition (Table 2, Figure 1E, and Supplementary Figure 1). The phosphorylated residues were plotted on TF with respect to the functional domains, TFR binding motif and iron binding sites (Supplementary Figure 1). The phosphorylated TF (P-TF) residues were not detected in the CaMKK2 KD neurons. Crystal structure of TF revealed that Ser381/389 and Thr392/393 residues overlapped with the TFR binding site (Figures 1E–G). In addition, immunoblotting based quantification revealed a significant reduction of total TF in the CaMKK2 KD neurons (Figure 1D). In some mass spectrometry spectrums, the fragmentation pattern could not resolve specific phosphorylated residues due to the presence of closely spaced Ser/Thr/Tyr residues and fragmentation between them did not occur (Table 2). Therefore, potential phosphorylated residues in the respective peptide fragment were separated by slash punctuation mark (Table 2). The mass spectrometry data is accessible in the Global Proteome machine (GPM) database (http://hs2.proteome.ca/tandem/thegpm_tandem.html) using respective GPM accession number (Table 2 and Figure 1C).

**Table 2:** List of phosphopeptides identified by LC-MS/MS analysis. log(e) for peptides represent the expectation value for the spectrum-to-sequence assignment.

**Log(e)**	**m+h (Da)**	**Start**	**Sequence (human serotransferrin)**	**End**	**Modifications**	**No of times observed in GPM**
**GPM ID: GPM10000002014 (Scrambled control)**						
−15	4348.937	381	pSVGKIEcCVSAETTEDcCIAKIoMNGEADAoMSL DGGFVYIAGK	420	S381+Phospho, C387+Carbamidomethyl, C396+Carbamidomethyl, M401+Oxidation, M408+Oxidation	2
−3.2	4412.908	381	pSVGKIECVpSAEpTpTEDcCIAKIMNGEADAoMSLDGGFVYIAGK	420	S381/389 & T392/393+Phospho, C387+C396 Carbamidomethyl, M408+Oxidation	2
−3.9	3364.386	391	dEpTpTEDcCIAKIoMNGEADAMpSL DGGFVYIAGK	420	T392/393+Phospho, S409+Phospho, E391+Dehydrated, C396+Carbamidomethyl, M401+Oxidation	815
−1.4	2337.942	490	INHcCRFDEFFpSEGcCAPGSK	508	C493+Carbamidomethyl, S500+Phospho, C503+Carbamidomethyl	9,565
**GPM ID: GPM10000002011 (Scrambled control)**						
−2.7	2785.36	251	RKPVDEpYKDcCHLAQVPSHTVVAR	273	Y257+Phospho, C260+Carbamidomethyl	105
−4.1	3689.642	333	pYLGpYEpYVTAIRNLREGTcCPEAPTDEcCKPVK	362	Y333/336/338+Phospho, C350+Carbamidomethyl, C358+Carbamidomethyl	0
−5.8	3364.386	391	dEpTpTEDcCIAKIoMNGEADAoMSLDGGFVYIAGK	420	E391+Dehydrated, T392/393+Phospho, C396+Carbamidomethyl, M401/408+Oxidation	815
−2.4	2337.942	490	INHcCRFDEFFpSEGcCAPGSK	508	C493+Carbamidomethyl, S500+Phospho, C503+Carbamidomethyl	9,565
−5.2	2621.123	509	KDpSpSLcCKLcCoMGSGLNLcCEPNNK	530	S511/512+Phospho, C514+Carbamidomethyl, C517+Carbamidomethyl, M518+Oxidation, C525+Carbamidomethyl	743
−1.4	2605.128	509	KDpSSLcCKLcCMGSGLNLcCEPNNK	530	S511+Phospho, C514+Carbamidomethyl, C517+Carbamidomethyl, C525+Carbamidomethyl	743
−2	2258.052	572	NLNEKDYELLcCLDGpTRKP	589	C582+Carbamidomethyl, T586+Phospho	3

*o, oxidation; c, carbamidomethyl; p, Phosphorylation; d, dehydration. The GPM ID numbers can be used to access MS-MS spectrum data at the Global Proteome Machine (GPM) database using the link: http://hs2.proteome.ca/tandem/thegpm_tandem.html*.

### Immunoblotting using anti-TF antibody revealed a significant reduction of P-TF (pH~3-4 fraction) in CaMKK2 KD DRG neurons

**Figure 2:**
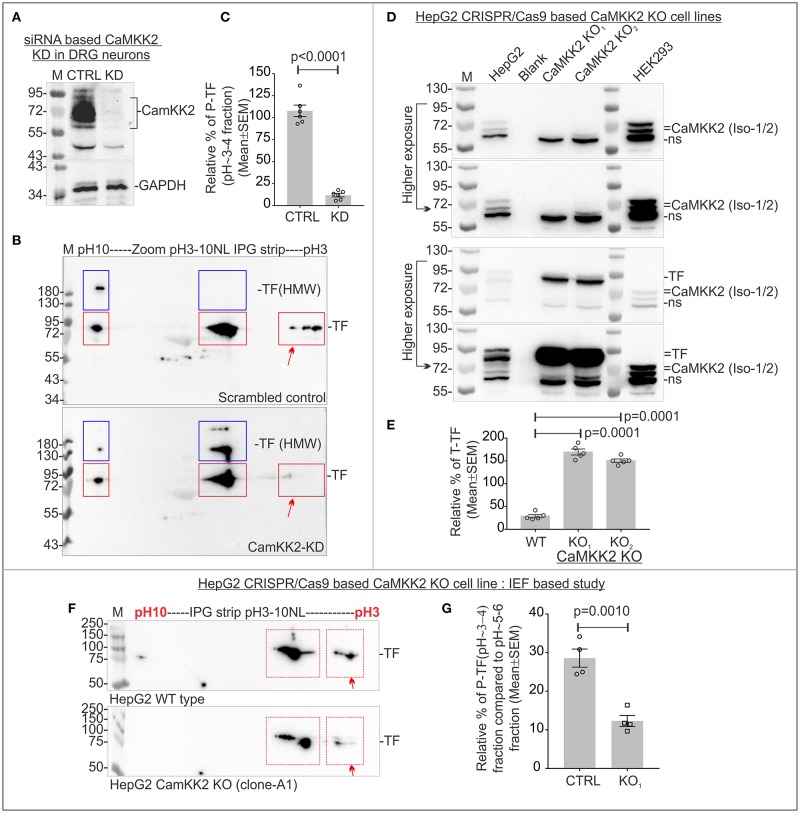
P-TF significantly reduced in the CaMKK2 KD cultured primary adult rat DRG neurons and multiple CaMKK2 KO human cell lines. **(A)** Immunoblots showing relative expression of CaMKK2, and GAPDH in CaMKK2 KD DRG neurons. CTRL, scrambled control; KD, knockdown. **(B)** Immunoblots showing charged fractions of TF in the DRG neurons. Red rectangles indicate pH/pI~3, ~5-6 and ~9-10 fractions of native TF. Blue rectangles indicate higher molecular weight (HMW) form of the TF. **(C)** Scatter plot showing relative percentage of P-TF. The percentage was normalized relative to the intensity of pH~9-10 fraction. *N* = 6 from 3 independent experiments. The *p*-value by *t*-test (unpaired). **(D)** Immunoblots showing expression of CaMKK2 and TF in CRISPR/Cas9 based CaMKK2 KO HepG2 cells and wild-type HEK293 cells. KO_1_ and KO_2_ represent independently selected CaMKK2 KO HepG2 clonal cell lines. Different exposure of the same blot was given to highlight bands at different intensities. **(E)** Scatter plot showing the relative amount of total TF. The percentage was calculated relative to GAPDH (not shown) intensity. *N* = 6 from 2 independent experiments. The *p*-value by *t*-test (unpaired). **(F)** Immunoblots showing charged fraction of the TF in the wild-type (WT) and CaMKK2 KO_1_ HepG2 cells. **(G)** Scatter plot showing the relative amount of P-TF. The relative intensity of the P-TF was calculated based on the intensity of pH~5-6 fraction. *N* = 4 from 4 independent experiments. The *p*-value by *t*-test (unpaired).

The molecular weight and pI of human/rat TF (SwissPort: P02787/P12346) is 77/76 kDa and 6.81/7.14, respectively. The defined neuronal culture media was supplemented with partially saturated (with iron) recombinant human TF. Therefore, cross-species mixture of TF was expected in the protein analysis. IEF/SDS-PAGE followed by immunoblotting using anti-TF antibody revealed that the charged fractions of TF differed between scrambled siRNA control and CaMKK2 KD DRG neurons (Figures 2A,B). TF appeared as 3 major charged fractions at pH~3-4, ~5-6, and ~9-10 (Figure 2B, red rectangles). Previous mass spectrometric analysis revealed that the pH~3-4 fractions corresponded to potential Ser/Tyr/Thr phosphorylated TF and therefore, hereafter designated as P-TF. Phosphorylated TF epitope specific antibody is not available, therefore, in the subsequent studies, the pH~3-4 fraction was quantified as a measure of P-TF. The relative amount of P-TF was significantly decreased in CaMKK2 KD neurons (Figures 2B,C, red arrow). This supported previous protein profiling based observation (Figure 1B, blue rectangles). The CaMKK2 KD DRG neurons exhibited the presence of additional ~130/>180 kDa focused spots in pH ~5-6 and pH~9-10 regions, whereas scrambled control cells exhibited >180 kDa spots in pH ~9-10 region only (Figure 2B, blue rectangles). The high molecular weight (HMW) forms may be due to PTMs that added additional molecular mass, for example, glycosylation. TF is known N-linked glycosylated at multiple residues (Satomi et al., 2004). Treatment with a deglycosylation mix (protein deglycosylation mix, NEB), containing all enzymes to remove N- and O-linked glycans caused the disappearance of the HMW TF indicating potential glycosylation (data not shown).

### P-TF was significantly reduced in the CRISPR/Cas9 based CAMKK2 KO HepG2 and HEK293 cells

**Figure 3:**
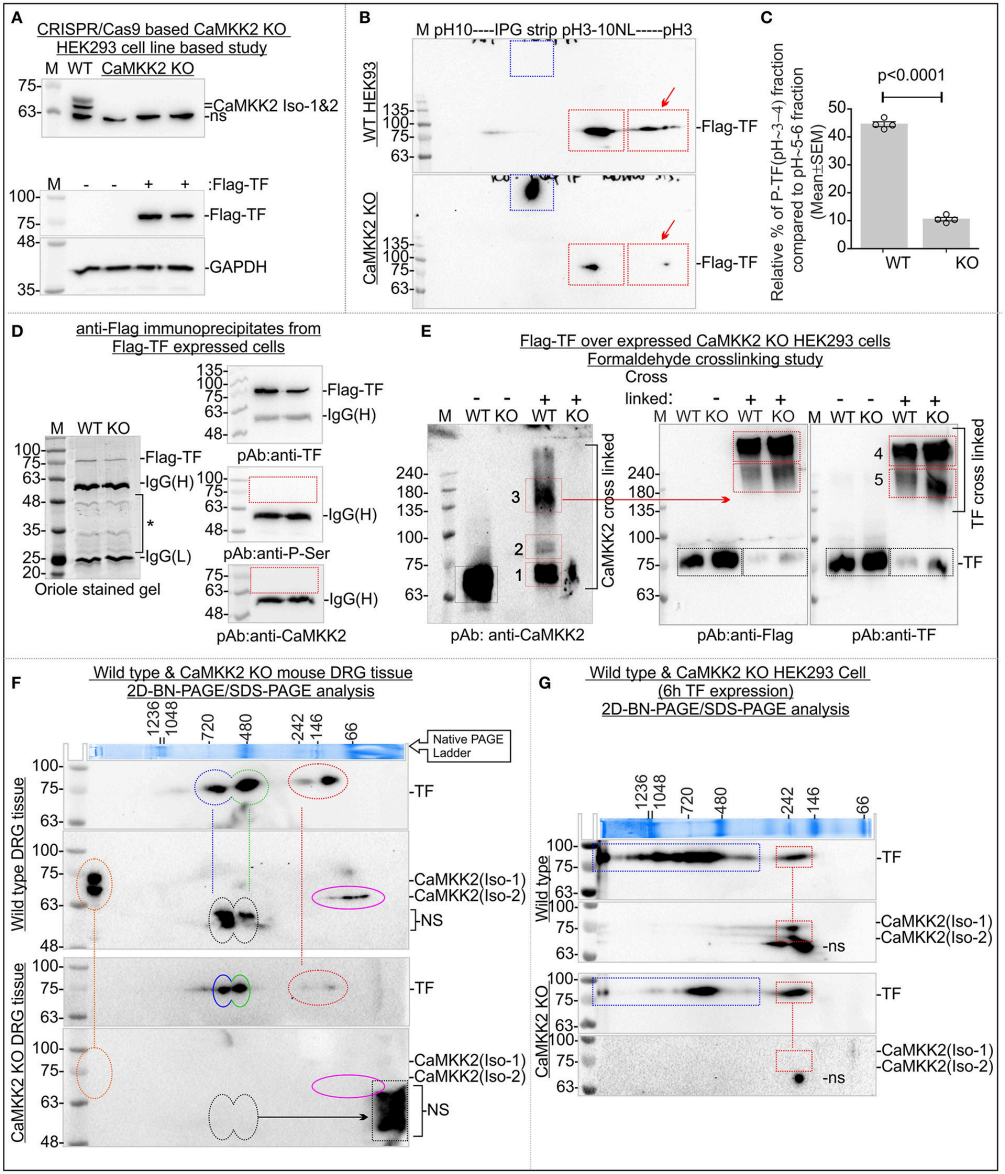
CaMKK2 is not directly associated with TF and loss of CaMKK2 affected TF associated protein complexes. **(A)** Immunoblot showing expression of CaMKK2 in the wild-type and three CRISPR/Cas9 based CaMKK2 KO HEK293 clonal cell lines selected independently (Top panel). Bottom panel: Flag-TF and GAPDH expression in HEK293 cells. **(B)** Immunoblots showing charged fractions of Flag-TF in the wild-type and CaMKK2 KO HEK293 cells. The red arrow represents P-TF. Blue square represents HMW TF. **(C)** Scatter plot showing the relative amount of P-TF. *N* = 4 from 4 independent experiments. The *p*-value by *t*-test (unpaired). **(D)** Immunoblots showing the presence of the TF and absence of the CaMKK2 in the Flag-TF immunoprecipitated fraction. The same protein fraction was also immunoblotted with the pan-P-ser specific antibody. **(E)** Immunoblots showing the relative shift of crosslinked CaMKK2 and TF in Flag-TF expressed wild-type and CaMKK2 KO HEK293 cells. The red squares represent the relative shift of the crosslinked proteins. Black Square represents native non-crosslinked form, shifted bands are numbered in Arabic numerals. **(F,G)** Immunoblots showing TF and CaMKK2 associated MPCs in mouse wild-type and CaMKK2 KO DRG tissues and Flag-TF expressed wild-type and CaMKK2 KO HEK293 cells. Dotted vertical lines indicate vertical alignment of the co-migrated protein complexes. Colored circles indicate MPCs. ns, Non-specific. The Coomassie stained gel strip on the top panel showing native page molecular weight ladder.

CRISPR/Cas9 based CaMKK2 KO HEK293 and HepG2 cell lines were generated to study the effect of CaMKK2 on TF phosphorylation. The HepG2 and HEK293 cell lines were derived from human hepatocellular carcinoma (liver in origin) (Aden et al., 1979) and embryonic kidney, respectively (Shaw et al., 2002). Human Protein Atlas (www.proteinatlas.org) based gene expression data revealed that HepG2 cells express TF but HEK293 cells do not express TF (Uhlen et al., 2005). Immunoblotting revealed that HepG2 strongly express TF whereas HEK293 cells do not express TF or expresses below the detection limit (Figures 2D, 3A). HEK293 and HepG2 cells expressed 2 isoforms (isoform 1 and 2) of CaMKK2 at ~60–75 kDa region and the relative expression of CaMKK2 differed between the cell lines (Figure 2D). In CaMKK2 gene ablation strategy, the CRISPR/Cas9 KO plasmids and CaMKK2 HDR plasmids were designed to cause targeted insertion of a reporter/selection cassette between exon 4 and 13 of the CaMKK2 gene which would effectively eliminate expression of all transcriptional isoforms. Immunoblotting revealed loss of expression of the CaMKK2 isoforms in 2 independently selected CaMKK2 KO HepG2 clonal cell lines (Figure 2D). Total TF level was significantly increased in the CaMKK2 KO HepG2 cell lines (Figures 2D,E). IEF/SDS-PAGE revealed 2 major charged fractions of TF in the pH~3-4 and ~5-6 regions in HepG2 cells (Figure 2F, red rectangles). The P-TF level was significantly decreased in the CaMKK2 KO HepG2 cells (Figures 2F,G, red arrow).

Immunoblotting revealed loss of CaMKK2 isoforms in the CaMKK2 KO HEK293 clonal cell lines (Figure 3A). HEK293 cells do not express TF or express it below the detection limit, therefore, Flag-tagged TF was expressed in the wild-type and CaMKK2 KO HEK293 cells to study the effect on TF phosphorylation (Figure 3A). IEF/SDS-PAGE revealed 2 major fractions of TF in the pH~3-4 and ~5-6 regions in HEK293 cells (Figure 3B). The P-TF level was significantly reduced in Flag-TF expressed CaMKK2 KO HEK293 cells compared to the wild-type (Figures 3B,C, Red arrow). In addition, Flag-TF appeared as a HMW fraction in the CaMKK2 KO HEK293 cells compared to the wild-type cells, as observed previously in CaMKK2 KD neurons (Figure 3B, Blue Square). This indicates difference in potential glycosylated form of TF.

### CaMKK2 is not associated with TF

In order to see if CaMKK2 is associated with TF, Flag-TF was immunoprecipitated from wild-type and CaMKK2 KO HEK293 cells and immunoblotted using anti-TF, anti-CaMKK2 and anti-pan-P-Ser specific antibodies (Figure 3D). The pan-P-Ser specific antibody was used because P-Ser is the abundant phosphorylation detected in the pH~3-4 fraction of TF. Protein gel staining, as well as immunoblotting, confirmed pull-down of Flag-TF (Figure 3D, left panel). However, CaMKK2 was not detected in the immunoprecipitated fraction of TF, neither any bands equivalent to P-TF(Ser) (Figure 3D, right panel).

The physical interaction between CaMKK2 and TF may be “hit and run” type with short residence time. Therefore, formaldehyde crosslinking followed by gel mobility shift assay (Klockenbusch and Kast, 2010) was used to study potential association between CaMKK2 and TF. Flag-TF was transiently expressed in the wild-type and CaMKK2 KO HEK293 cells and crosslinked using formaldehyde. Formaldehyde is the shortest cross-linker with a crosslinking distance of 2.3–2.7Å (Klockenbusch and Kast, 2010). The non-crosslinked and crosslinked proteins in Flag-TF expressed wild-type and CaMKK2 KO cells were resolved by SDS-PAGE and immunoblotted (Figure 3E). Native non-crosslinked CaMKK2 isoforms appeared within 63–75 kDa (black square) whereas crosslinked CaMKK2 appeared as 3 major bands at ~75, ~80–90, and ~135–180 kDa (red squares, numbered 1–3) (Figure 3E, left panel). The corresponding CaMKK2 bands were absent in the KO cells as expected (Figure 3E). Non-crosslinked Flag-TF appeared at 75 kDa (black square) whereas crosslinked Flag-TF appeared as 2 major shifted bands at ~180–240 and >240 kDa (red squares, numbered 4–5) (Figure 3E). The formaldehyde crosslinked TF associated bands displayed a comparative difference between the wild-type and CaMKK2 KO cells (Figure 3D, compex-5) indicating a difference in the association with interacting proteins. The relative position of the native CaMKK2 and Flag-TF shifted to multiple HMW bands in the crosslinked samples which suggested a shift in mass due to crosslinking with interacting proteins, however, the absence of any overlap between CaMKK2 and TF associated bands indicates the absence of any direct interaction.

BN-PAGE/SDS-PAGE was used to study the dynamics of TF and CaMKK2 associated protein complexes (Figure 3F). In BN-PAGE/SDS-PAGE, the first dimension native BN-PAGE separates the multiprotein complexes (MPCs) and the 2nd dimension denatured SDS-PAGE separates interacting protein components in the MPC which appears on a vertical line (Sabbir et al., 2016). BN-PAGE/SDS-PAGE revealed that TF formed 4 major MPCs at ~720, ~480, ~242, and ~100 kDa in the wild-type DRG tissues (Figure 3F; blue, green, and red circles). CaMKK2 isoforms formed MPCs at >1,200 and ~66 kDa (Figure 3F, orange and pink circles). The CaMKK2 MPCs were absent in the CaMKK2 KO mouse DRG tissues (Figure 3F). The absence of any vertical alignment of the native CaMKK2 and TF associated MPCs indicates that these proteins do not associate directly in a stable protein complex. The relative position of the ~720 and ~480 kDa TF MPCs shifted (blue and green circle) and the ~242 and ~100 kDa TF MPCs (red circle) disappeared in the CaMKK2 KO DRG tissues (Figure 3F). This indicates that the dynamic appearance of TF associated MPCs depends on CaMKK2.

The dynamics of TF and CaMKK2 associated MPCs were also studied in Flag-TF overexpressing wild-type and CaMKK2 KO HEK293 cells (Figure 3G). In wild-type HEK293 cells, TF appeared as multiple MPCs at >1,200, ~1,200–480, and 242 kDa (blue and red rectangles) and CaMKK2 isoforms appeared at 242 kDa (red rectangle), respectively (Figure 3G). This difference indicates potential cell type-specific difference in the interacting proteins. In CaMKK2 KO HEK293 cells, TF associated >1,200 and ~1,200–480 kDa MPCs were comparatively decreased and CaMKK2 isoform associated MPC disappeared (Figure 3G). In wild-type HEK293 cells, TF and CaMKK2 isoforms both vertically aligned at 242 kDa, but the 242kDa TF MPC was not shifted in its relative position in CaMKK2 KO condition which indicates that these proteins are not associated in the same complex though they appeared vertically aligned in the BN-PAGE.

### Loss of CaMKK2 leads to defective TF uptake and internalization

**Figure 4:**
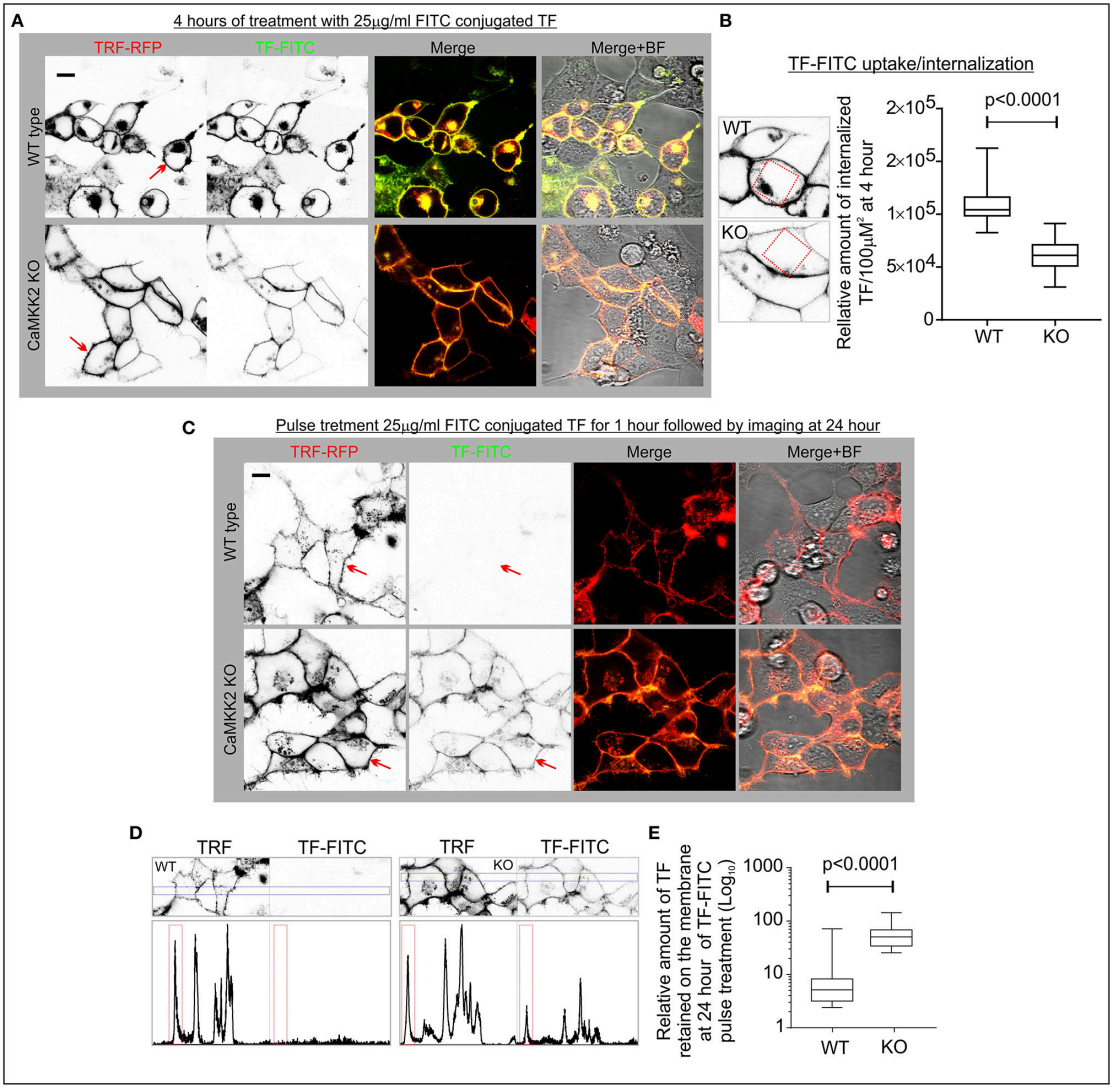
Aberrant TF uptake and trafficking in the CaMKK2 KO HEK293 cells. **(A)** Live confocal fluorescent images showing localization of FITC-TF and RFP-TFR in the wild-type and CaMKK2 KO cells. The red arrow indicates the membrane localization of TFR. **(B)** Whisker plot showing relative intensity of the internalized TF. Red square represents 100 μM^2^ intracellular area used to measure the relative intensity of internalized TF. The *p*-value calculated by *t*-test (unpaired). **(C)** Live confocal fluorescent images showing membrane-bound FITC-TF after 24 h of incubation in serum-free media following pulse treatment with 25 μg/ml FITC-TF for 4 h. The red arrow indicates the membrane localization of TFR and FITC-TF. **(D)** The red and green channel images presented in **(C)** were stitched together to create an intensity profile plot for the blue rectangular marked area. The profile plot was then used for calculating the relative percentage of membrane-bound FITC-TF. The peak area in the red rectangles represents membrane-associated TFR and FITC-TF. All confocal (Zeiss, LSM510) images were taken using the same image acquisition parameters for the wild-type and CaMKK2 KO cells. The image analysis was performed using ImageJ software. **(E)** Whisker plot showing relative percentage of TF associated with the membrane. *N* = 50 from 2 independent experiments. The *p*-value calculated by *t*-test (unpaired).

RFP tagged TFR was expressed in the wild-type and CaMKK2 KO HEK293 cells and FITC-conjugated TF (FITC-TF) uptake and internalization were studied to see the effect of CaMKK2 loss (Figure 4). RFP-TFR was localized in the cell membranes (Figure 4A, red arrow). Internalized FITC-TF associated vesicular structures were significantly reduced in the CaMKK2 KO cells compared to the wild-type cells following 4 h of 25 μg/ml FITC-TF treatment indicating reduced internalization (Figures 4A,B, red square). In addition, pulse treatment with 25 μg/ml FITC-TF for 4 h followed by replacement of the extracellular fluid with serum-free medium and subsequent 24 h of incubation revealed significant retention of membrane-bound FITC-TF in CaMKK2 KO cells compared to the wild-type cells (Figures 4C,D, red arrows, and squares). Overall, this indicates that loss of CaMKK2 significantly altered TF uptake and internalization which associated with reduced P-TF in CaMKK2 KO HEK293 cells.

### Loss of CaMKK2 leads to abnormal Ca^2+^ release response

In order to see if a loss of CaMKK2 leads to the abnormal intracellular Ca^2+^ response, we treated wild-type and CaMKK2 KO HEK293 cells with 10 μM muscarine and measured the temporal dynamics of intracellular Ca^2+^ using cell-permeant fluorescent calcium indicator (Fluo-4). The HEK293 cells endogenously express muscarinic receptors and exhibit G-protein coupled intracellular Ca^2+^ release response upon muscarine treatment (Hussmann et al., 2011). Muscarine treatment elicited an immediate spike (within seconds) followed by a sustained elevation in intracellular Ca^2+^ in the wild-type cells in presence of Ca^2+^ in the extracellular environment (Supplementary Figure 2A, blue line). In CaMKK2 KO HEK293 cells, muscarine treatment caused a similar spike followed by a drop and then an exponential increase in intracellular Ca^2+^ (Supplementary Figure 2A, red line). At 3.5 min of muscarine treatment, the intracellular Ca^2+^ release was significantly lower in CaMKK2 KO cells compared to the wild-type cells which was reversed at 10 min of treatment (Supplementary Figures 2B,C). Thus, the dynamics of muscarinic receptor ligand-induced Ca^2+^ response was altered by loss of CaMKK2. Interestingly, in absence of Ca^2+^ in the extracellular media, CaMKK2 KO cells exhibited a significantly reduced amount of Ca^2+^ release compared to the wild-type cells (Supplementary Figures 2E,F). These indicate that CaMKK2 loss may affect plasma membrane/ endoplasmic reticulum-bound ion channels as well as Ca^2+^ buffer proteins.

### Loss of CaMKK2 ubiquitously and significantly reduced P-TF but total TF was altered in a tissue-specific manner

**Figure 5:**
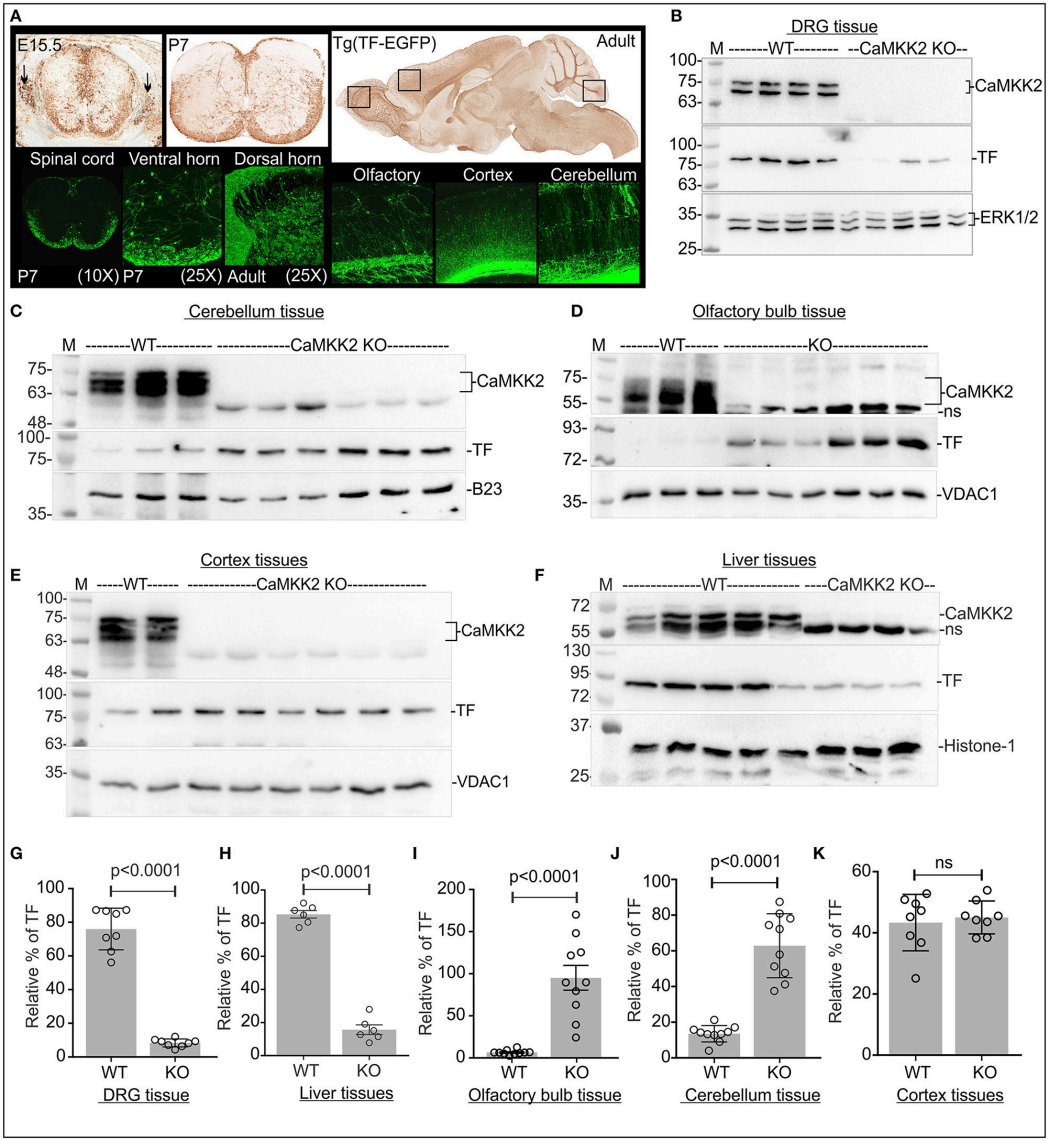
Loss of CaMKK2 differentially affected relative abundance of total TF in a tissue-specific manner in the CaMKK2 KO mouse. **(A)** TF promoter-trapped GFP reporter expression in adult, postnatal (P7) and embryonic 15.5 stage spinal cord, DRGs and adult brain tissues (founder line: IF181). The images were obtained from The Gene Expression Nervous System Atlas (GENSAT) Project, NINDS Contracts N01NS02331 & HHSN271200723701C to The Rockefeller University (New York, NY). Top panel: GFP-immunostaining of paraffin-embedded sections. Bottom panel: GFP epifluorescence in cryomicrotome sections. **(B–F)** Immunoblots showing expression of CaMKK2, TF, and ERK1/2, VDAC1, histone-1 (H1), nucleophosmin (B-23), and GAPDH in different adult mouse tissues. **(G–K)** Scatter plot showing the relative amount of TF in different adult mouse tissues. DRG tissue: ERK1/2 used for normalization, *N* = 8 replicates from 4 mice in each category. Olfactory/cerebellum tissues: GAPDH/B23 used, respectively, for normalization. *N* = 10/5 replicates from 3 KO and wild-type mice, respectively. Cortex/Liver tissues: VDAC1/H1 used, respectively, for normalization, *N* = 8/6 replicates from 3 CaMKK2 KO and wild-type mice, respectively. The *p*-value by *t*-test (unpaired).

TF promoter-trapped GFP reporter expression in the mouse central and peripheral nervous system (CNS and PNS) (GENSAT: Gene Expression Nervous System Atlas project, Rockefeller University, New York, USA) revealed that TF is expressed in the neurons of the spinal cord, olfactory bulb, cortex and cerebellum (Figure 5A). Immunoblotting revealed expression of CaMKK2 isoforms equivalent to 75 and 70 kDa proteins in the DRG (b), cerebellum (c), olfactory bulb (d), cortex (e) and liver (f) tissues (Figure 5). In CaMKK2 KO mice, protein bands corresponding to CaMKK2 isoforms were absent as expected (Figures 5B–F). The relative amount of TF was significantly decreased in CaMKK2 KO DRGs (g) and liver (k), significantly increased in the olfactory bulb (i) and cerebellum (j), and remained unaltered in cortex (k) tissues compared to the wild-type mice (Figure 5). This indicates that the relative abundance of TF was regulated by CaMKK2 in a tissue-specific manner.

**Figure 6:**
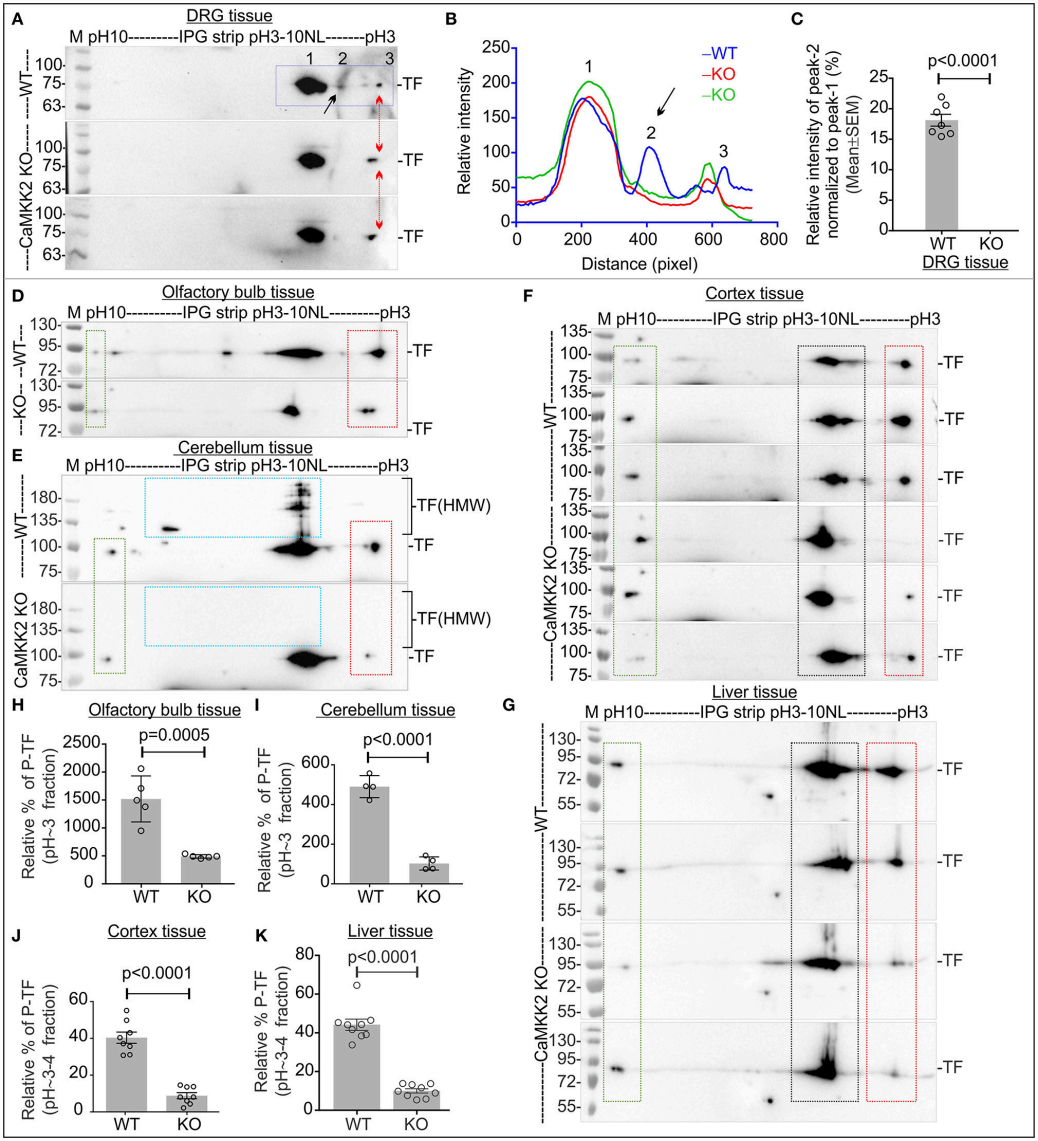
P-TF was ubiquitously and significantly reduced in the CaMKK2 KO mice. **(A,D–G)** Immunoblots showing charged fractions of TF. Rectangle areas represent charged fraction of TF at different pH/pIs. The red arrow/red square indicates altered P-TF (pH~3-4 fractions). The green rectangle area represents TF fraction which was used to normalize the intensity of the pH~3-4 fractions in the red square area. **(B)** Intensity profile of the focused spots presented in the immunoblots in **(A)**, blue squared area. The spots are marked with Arabic numerals and spot 2 was quantified. **(C)** Scatter plot showing the relative intensity of spot 2 in pH~3-4 fractions of TF. *N* = 7 replicates from 3 mice in each category. The *p*-value by *t*-test (unpaired). **(H–K)** Scatter plot showing the relative amount of P-TF (pH~-4 fractions) in olfactory bulb, cerebellum, cortex and liver tissues, respectively. *N* = 5/4/7/8 replicates from 3 to 4 KO and wild-type mice, respectively. The *p*-value by *t*-test (unpaired).

IEF/SDS-PAGE revealed that TF appeared as 2 charged fractions in pH~3-4 and ~5-6 regions in the DRG tissues (a) and 3 charged fractions in pH~3-4, ~5-6, and ~9-10 regions in the olfactory bulb (d), cerebellum (e), cortex (f), and liver (g) tissues (Figure 6). In DRG tissues, the P-TF focused spot in pH~3-4 fraction (peak profile numbered 2 in Figure 6B, black arrow), was quantified and found significantly reduced in the CaMKK2 KO DRGs compared to the wild-type (Figure 6C). The relative amount of P-TF was significantly reduced in the CaMKK2 KO mice olfactory bulb (h), cerebellum (i), cortex (j), and liver (k) tissues (Figure 6, red rectangles). This indicates that loss of CaMKK2 ubiquitously reduced TF phosphorylation.

### Altered CaMKK2 and significantly reduced P-TF is associated with early and late-stage of AD in 3xTg-AD mice

The triple transgenic 3xTg-AD mice were used to study the charged fractions of CaMKK2 and TF during the progression of AD. Only female 3xTg-AD mouse mice were used to avoid gender reported differences in neuropathology and behavior (Hirata-Fukae et al., 2008; Gimenez-Llort et al., 2010; Garcia-Mesa et al., 2011; Hebda-Bauer et al., 2013). A progressive increase in the Aβ peptide deposition was detected in some brain regions of 3xTg-AD mice as early as 3–4 months (Oddo et al., 2003). Synaptic transmission and long-term potentiation were impaired at 6 months in 3xTg AD mice (Oddo et al., 2003). Conformationally altered and hyperphosphorylated tau was detected in the hippocampus of 3xTg-AD mice at 12–15 months (Oddo et al., 2003). Therefore, 6 months and 14 months were considered as an early and late stage of AD in the 3xTg-AD mouse model and studied.

**Figure 7:**
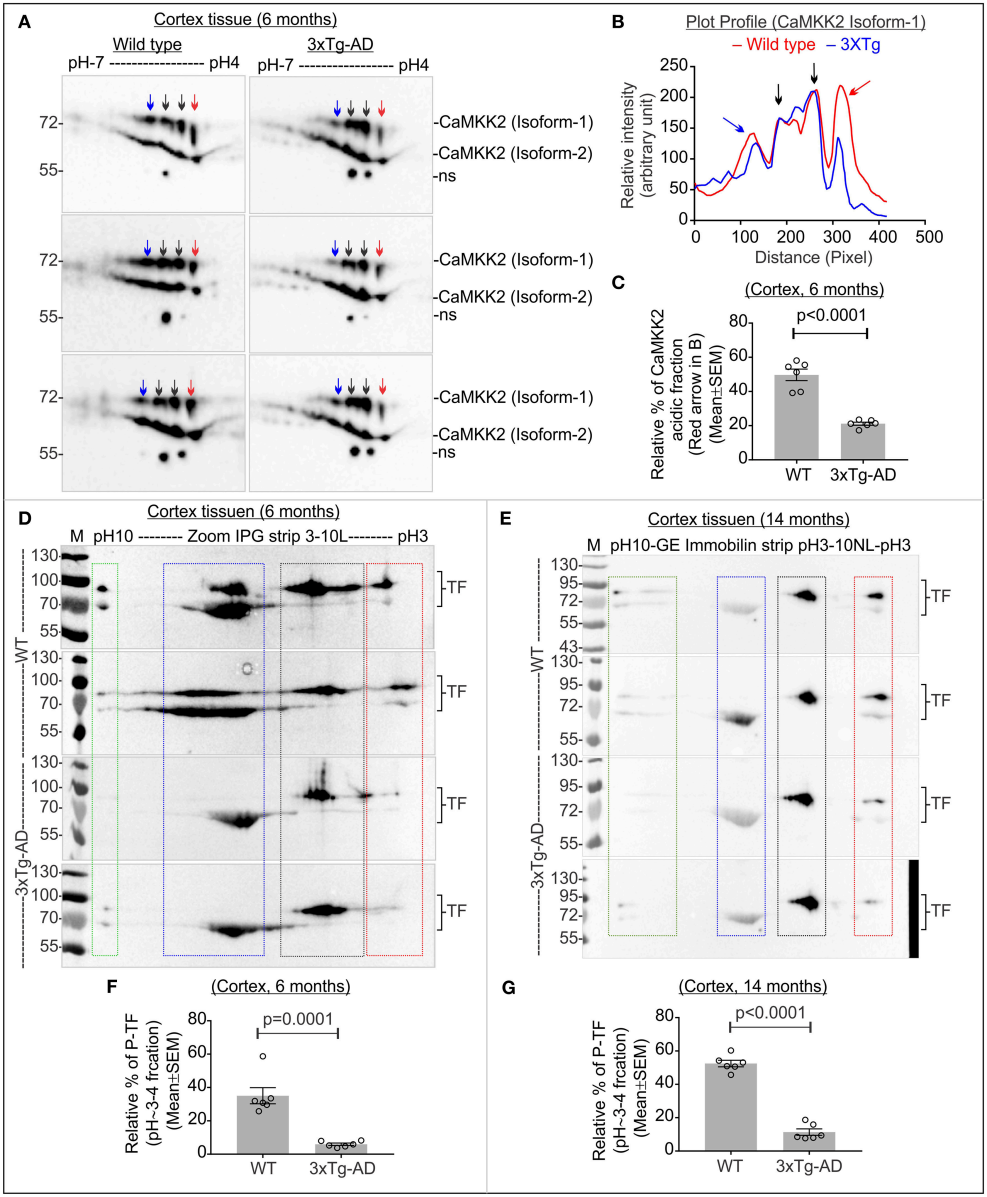
Altered CaMKK2 and reduced P-TF in 3xTg-AD mice. **(A)** Immunoblot showing charged fractions of CaMKK2 isoform 1 and 2 in 6 months' old wild-type and 3xTg-AD mice. Colored arrows indicate different charged fractions. Linear pH 4-7 IPG strips were used to resolve closely spaced CaMKK2 charged fractions. **(B)** Plot profile showing relative intensity of the focused CaMKK2 isoform-1 spots. **(C)** Scatter plot showing relative percentage of the comparatively more negative charged fraction (red arrow) of CaMKK2 isoform-1. The spots marked with black arrows were used for normalization because their intensity was not altered in different categories. **(D,E)** Immunoblots showing charged fractions of TF in 6 and 14 months old 3xTg-AD mice cortex tissues. The colored rectangle indicates different charged fractions. **(F,G)** Scatter plot showing relative intensities of the P-TF (red rectangle). The intensities of pH~5-6 fractions were used for normalization. *N* = 6 replicates from 3 mice in each category. The *p*-value by *t*-test (unpaired).

The molecular weight and pI of the mouse CaMKK2 isoforms (transcript ID: ENSMUST00000111668.7 and ENSMUST00000200109.4, respectively) were theoretically predicted as 73/59kDa and 5.27/5.31, respectively by ExPASy-Compute pI/MW tool (Gasteiger et al., 2003). IEF/SDS-PAGE of age and sex-matched early 3xTg-AD and wild-type cortex tissues revealed the presence of ~73 and ~59 kDa CaMKK2 proteins corresponding to isoform-1 and -2, respectively (Figure 7A). Plot profile of the focused CaMKK2 isoform 1 spots (Figure 7B) revealed a comparative difference between the wild-type and 3xTg AD mice in the intensity of the red arrow marked peak which is more negative shifted. IEF/SDS-PAGE study of the immunoprecipitated CaMKK2 from mammalian cells by Green et al. showed that the multiple CaMKK2 focused spots as observed in this study were positive for anti-P-Ser antibody and mutation of S129A, S133A, and S137A lead to the disappearance of the majority of the spots (Green et al., 2011). Validated P-CaMKK2 antibodies are not available. Comparatively more negative pI/pH shifted fraction of the CaMKK2 was considered as potentially phosphorylated and relative quantification revealed significant reduction of the CaMKK2 negative charged fraction (red arrow marked fraction) in the early 3xTg-AD cortex tissues compared to the wild-type mice (Figures 7A–C). TF in the early and late 3xTg-AD cortex tissues appeared as 4 major charged fractions in pH~10, ~7-8, ~5-6, and ~3-4 regions (Figures 7D,E, colored rectangles). P-TF was significantly decreased in both early and late 3xTg-AD cortex tissues compared to the wild-type mice (Figures 7D–G, red rectangles).

### Altered TF MPCs were associated with 3xTg-AD mouse cortex and hippocampus

**Figure 8:**
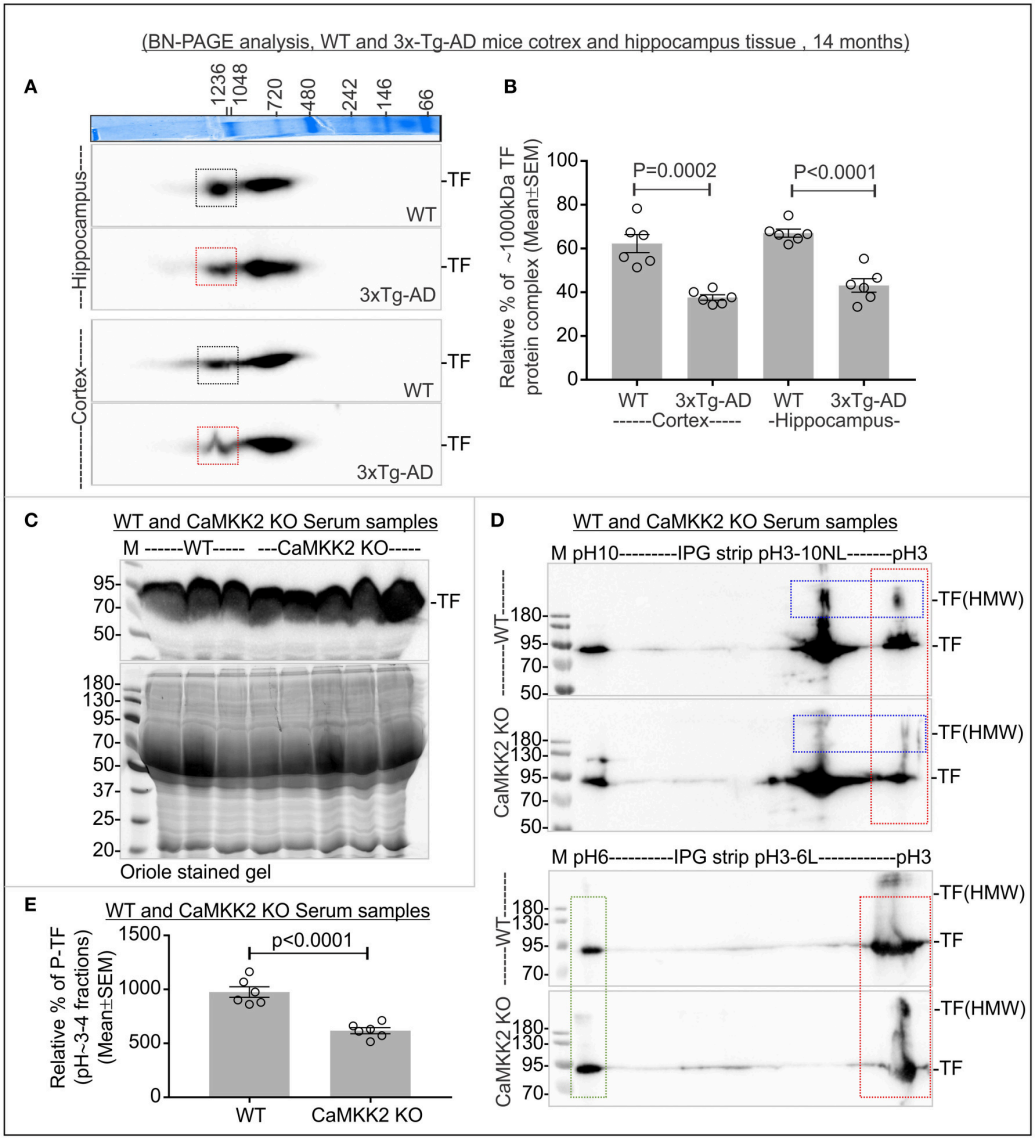
Altered TF associated protein complexes in 3xTg-AD mice cortex and hippocampus, and significantly reduced P-TF in CaMKK2 KO mice serum. **(A)** Immunoblots showing TF associated MPCs in the cortex and hippocampus tissues from 14 months old wild-type and 3xTg-AD mice. The Coomassie stained gel strip on the top panel showing native page molecular weight ladder. Colored squares represent a change in the relative intensity. **(B)** Scatter plot showing relative intensities of ~1,000 kDa TF associated MPC. The intensity of the ~720 kDa complex was used for normalization. **(C)** Immunoblot showing total TF level in the serum (top panel). Bottom panels: SDS-PAGE gel stained with Oriole to show total protein loading. **(D)** Immunoblots showing charged fractions of TF in the serum. Proteins were focused on IPG pH3-10NL strips (top panel) and IPG pH3-6L strips (bottom panel), respectively. The red dotted rectangle indicates P-TF (pH~3-4 fractions). Blue dotted rectangles indicate HMW TF. **(E)** Scatter plot showing relative abundance of P-TF in serum. The pH~3-4 fractions were normalized on the basis of pH~6 fraction intensities (green rectangle) in IPG pH3-6L strips. *N* = 6 replicates from 3 mice in all experiments. The *p*-value by *t*-test (unpaired).

TF appeared as 2 MPCs at ~1,000 and ~720 kDa in 14 months old mice cortex and hippocampus tissues (Figure 8A). The relative abundance of ~1,000 kDa TF MPC was significantly decreased in both the hippocampus and cortex of the late-stage 3xTg-AD mice (Figures 8A,B, red rectangles). This indicates that the altered negative charged fraction of CaMKK2 in 3xTg-AD mice brain tissues is associated with decreased P-TF which affected the dynamics of TF associated protein complexes.

### P-TF was significantly reduced in the serum of CaMKK2 KO and 3xTg-AD

**Figure 9:**
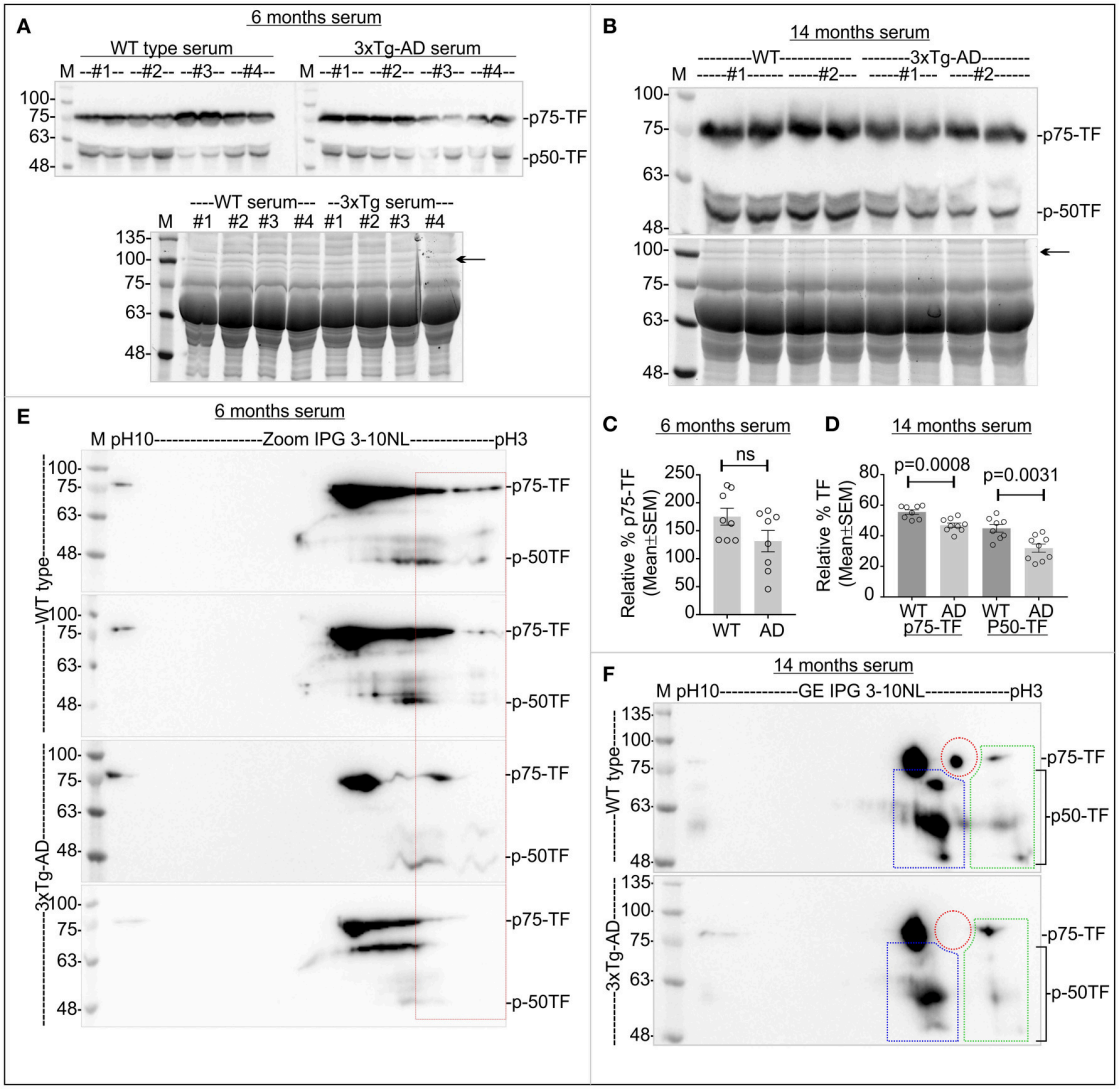
Relative abundance of total TF and P-TF in the serum samples from early and late 3xTg-AD and age-matched control mice. **(A,B)** Top Panels: Immunoblots showing total TF level in the serum. Bottom panels: SDS-PAGE gel stained with Oriole to show total protein loading. Black arrows indicate the band used for normalization of total TF in the serum. **(C,D)** Scatter plot showing relative abundance of TF in the serum. *N* = 8 (2 replicates from 4 mice in each category. The *p*-value by *t*-test (unpaired). **(E,F)** Immunoblots showing charged fractions of TF. Red dotted area indicates P-TF (pH~3-4 fractions). Colored dotted rectangles in **(F)** indicate different charged fractions of the TF that also exhibited the marked difference between wild-type 3xTg-AD mice.

The relative abundance of TF remained comparatively unaltered in CaMKK2 KO serum (Figure 8C). However, the relative level of P-TF was significantly reduced in CaMKK2 KO serum (Figures 8D,E, red rectangles). This indicates that loss of CaMKK2 decreased serum P-TF level which may serve as a potential biomarker for AD. TF in 3xTg-AD serum appeared as 75 and 50 kDa proteins (p75-TF and p50-TF) (Figure 9A). The relative abundance of TF in early-stage 3xTg-AD mice was not significantly altered (Figures 9A,C), but in the late-stage 3xTg-AD mice, serum TF level was significantly decreased compared to the wild-type (Figures 9B,D). IEF/SDS-PAGE revealed comparatively decreased P-TF in the serum of both early and late 3xTg AD compared to the wild-type (Figures 9E,F, red rectangles). This indicates that the P-TF profile, not the total TF level in the serum reflect the physiological condition of AD in 3xTg-AD mice.

### The relative abundance P-TF was altered in the CSF and serum from early and late-onset human postmortem AD patients

**Figure 10:**
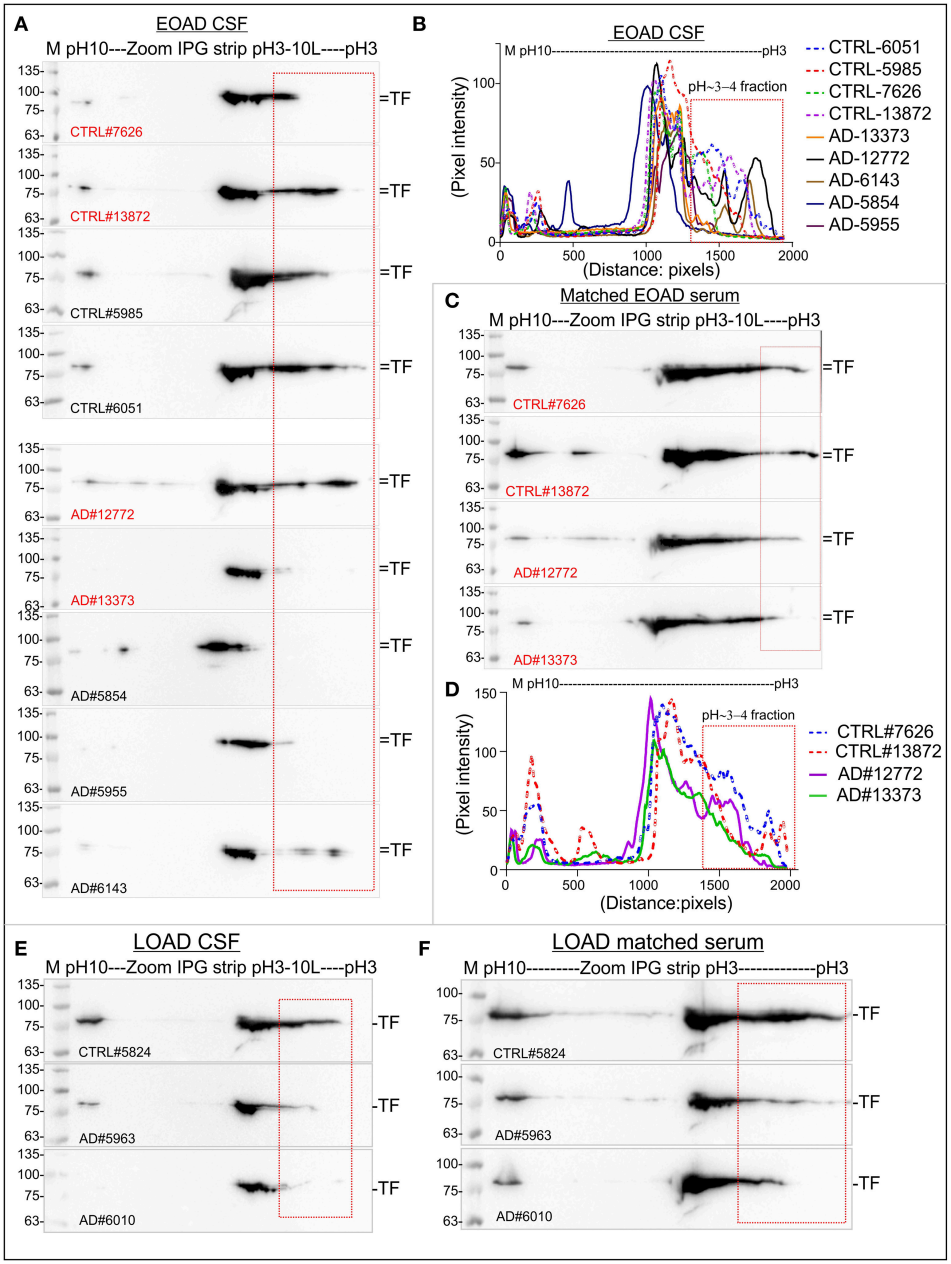
Relative abundance of P-TF reduced in the CSF and serum of postmortem human EOAD and LOAD patients compared to the controls. **(A,C,E,F)** Immunoblots showing charged fractions of TF in the CSF and serum samples of postmortem human EOAD and LOAD patients and unaffected controls. **(B,D)** plot profile of the immunoblots presented in **(A,C)**. The plot profile was created using ImageJ. Red marked are represents P-TF (pH~3-4 fractions). Detailed sample information is given in Table 3. The Red highlighted samples represent matched serum and CSF collected from same individual.

Total TF and the P-TF level were analyzed in the postmortem human CSF and serum samples from EOAD patients (Table 3). Matched Serum and CSF from the same individual was available for 2 age-matched EOAD and 2 unaffected control patients (Table 3). Matched serum and CSF samples were available from 3 LOAD and 1 unaffected control patients (Table 3). Total TF was significantly reduced in the CSF from EOAD patients compared to the age-matched unaffected controls (Supplementary Figures 3A,D). IEF/SDS-PAGE of the CSF samples from the same group of EOAD patients revealed comparative reduction/loss of P-TF compared to the unaffected control, except one patient (patient ID: 12772, Table 3) who was diagnosed with both AD and schizophrenia (Figures 10A,B, red rectangles). Matched serum from 2 EOAD patients (ID: 12772 and 13373) exhibited unaltered total TF level (Supplementary Figures 3C,F) but the P-TF level was reduced compared to the age-matched unaffected controls (Figures 10C,D, red rectangles). In addition, serum TF profile in these 2 EOAD patients resembled the CSF TF profile in terms of reduced P-TF level. This indicates that serum total TF level was not predictive of AD but the P-TF profile predicted diseased state. This supports P-TF (pH~3-4 fractions) profile as a potential biomarker for early-onset AD.

**Table 3:** Postmortem human AD and age matched unaffected control patient sample information.

**Patient ID**	**Fluid**	**Disorder**	**Age (years)**	**Sex**
**EOAD samples (**<**65 years)**				
7626	CSF & Serum	Unaffected control	57	Male
13872	CSF & Serum	Unaffected control	57	Male
12772	CSF & Serum	Alzheimer's disease	52	Male
13373	CSF & Serum	Alzheimer's disease	59	Male
5854	CSF	Alzheimer disease	56	Female
5955	CSF	Alzheimer disease; amyotrophic lateral sclerosis	58	Male
6143	CSF	Alzheimer disease; schizophrenia	59	Female
5985	CSF	Unaffected control	55	Male
6051	CSF	Unaffected control	52	Male
**LOAD samples (**>**65 years)**				
5824	CSF & Serum	Unaffected control	74	Male
5963	CSF & Serum	Alzheimer disease	76	Male
6010	CSF & Serum	Alzheimer disease	78	Female
6078	CSF & Serum	Alzheimer disease	74	Male

Total TF was comparatively reduced in the CSF of LOAD patients compared to the unaffected control but the serum TF level remained unaltered (Supplementary Figures 3G,H). IEF/SDS-PAGE revealed a comparative reduction of P-TF in both serum and CSF from the LOAD patients compared to the age-matched unaffected control (Figures 10E,F). This indicates that the P-TF profile in both serum and CSF may serve as a potential biomarker for the late-onset AD.

## Discussion

This study reports that the relative amount of phosphorylated TF corresponding to an acidic fraction of pI/pH~3-4 may serve as a potential biomarker for AD. Conceptualization of this study was based on the initial proteomics and mass spectrometry-based finding that KD of CaMKK2 in the neuronal cells significantly reduced pH~3-4 fraction of TF containing potentially phosphorylated Ser/Thr/Tyr residues. This basic finding was then extensively validated using multiple CaMKK2 KO human cell lines as well as CaMKK2 KD cultured primary adult rat DRG neurons. In addition, it has been shown that loss of CaMKK2 disrupted intracellular TF uptake and increased the membrane-associated fraction of TF. This provided a mechanistic explanation for the aberrant TF turnover in the brain and liver tissues due to the defective TF trafficking under CaMKK2 loss of function condition. Dysregulated Ca^2+^/CaMKK2 or its upstream kinases in the AD has been reported (Supnet and Bezprozvanny, 2010; Wang et al., 2017). Therefore, one of the logical outcomes of this study was to explore the possibility that altered CaMKK2 in the AD brain due to dysregulated Ca^2+^ may lead to the secretion of hallmark P-TF in the serum which in turn may serve as a potential biomarker for AD. Interestingly, the relative abundance of TF was significantly increased or decreased in a tissue-specific manner but the P-TF level was ubiquitously decreased in the CaMKK2 KO mice. Aberrant CaMKK2 in the early and late stage of the disease in 3xTg-AD mice associated with the reduction of P-TF in the cortex and significant reduction of TF associated MPCs in both cortex and hippocampus. Serum P-TF was significantly reduced in the CaMKK2 KO mice and comparatively reduced in the early and late-stage 3xTg-AD mice which further supported the potential of P-TF as a serum-based biomarker for AD. This led to the analysis of P-TF profile in postmortem human CSF and serum samples. Analysis of the CSF and serum from postmortem AD patients revealed a comparative reduction of P-TF in both EOAD and LOAD patients compared to the unaffected control which associated with the disease condition. This supported that P-TF (pH~3-4 fraction) profile may serve as a potential biomarker for AD. In addition, this study is the first report linking abnormal CaMKK2 with TF trafficking and turnover which may provide a novel insight into the neurodegeneration process.

Does CaMKK2 directly phosphorylate TF? Chemical crosslinking study, immunoprecipitation and native MPC analysis provided an indication that CaMKK2 may not be the kinase directly associated with TF and therefore, may not be catalytically involved in phosphorylation. The pan-phospho serine specific antibody was not able to recognize the P-TF(Ser) epitopes in the immunoprecipitated Flag-TF however, it recognized P-Ser residues in the monoclonal immunoglobulins (heavy chains) used for immunoprecipitation. Mouse Ig heavy chains are frequently phosphorylated at multiple Ser residues (www.phosphositeplus.org) (Zhong and Wright, 2013; Hornbeck et al., 2015). TF phosphorylation-state specific antibodies are required to study the observed complex phosphorylation patterns. The phosphorylated peptides identified in this study will provide a guideline for future epitope selection. In addition, the presence of potential phosphorylated Tyr residues suggested that a tyrosine kinase may be involved. CaMKK2 has been known to regulate tyrosine kinases, for example, loss of CaMKK2 uncoupled agonist-stimulated Toll-like receptor 4 (TLR4) from activation of protein tyrosine kinase 2 (PYK2) (Racioppi et al., 2012). CaMKK2 kinase activity assay using TF as a substrate is another experimental approach to address this question. However, success in such experimental approach depends on identifying the specific CaMKK2 isoform responsible for TF phosphorylation as well as understanding the requirement for specific CaM component integral for such kinase activity, which is the scope for future studies.

The function of the potential novel P-TF residues are not known but bioinformatics analysis suggested some important features. Majority of the P-TF residues have surface accessibility which indicates functional relevance (Supplementary Figure 4A). The Tyr257/333/338, Ser381/389/409/511, and Thr586 residues are conserved between different mammalian species (Supplementary Figure 1A). The Ser381/389 and Thr392/393 residues positioned within the conserved TFR binding site identified by cryo-electron microscopy (residues 349-372) (Cheng et al., 2004), radiation footprinting (residues 381-401) (Liu et al., 2003; Xu et al., 2005) and epitope mapping (residues 365-401) (Teh et al., 2005) (Figures 1F,G, Supplementary Figures 1A, 4A). Loss of phosphorylation at Ser381/389 and Thr392/393 may affect interaction of TF with TFR (Wally et al., 2006). P-TF(T392/393) has been previously detected in mass spectrometric analysis of cytoskeleton-associated proteome in HeLa cells (GPM ID: GPM70110006894) (Ozlu et al., 2010). The Ser409 residue is located in close proximity to one of the iron binding residue (Asp411) in the C-lobe of TF (Yang et al., 2012) and conserved in different mammalian species and (Supplementary Figure 1A). High stringency protein motifs search by the ScanSite “MotifScan” module function (Obenauer et al., 2003) revealed a plurality of short motifs putatively concerned with protein interactions and signal transduction molecules, including two SH2 target sequences, a tyrosine kinase, three acidophilic kinase, a Ser/Thr kinase, and a proline-dependent Ser/Thr kinase binding sites, as well as consensus binding sites for 3-Phosphoinositide-Dependent Protein Kinase 1 (PDPK1 or alternatively PDK1), overlapped the P-TF residues (Supplementary Figures 4B,C). For example, the Tyr333/336/338 residues overlapped with potential PDPK1 binding motif (Supplementary Figures 1A, 4B,C). The P-TF (Tyr333) residue in the PDPK1 binding motif (^332^ M_p_YLGYEYVTAIR^343^, GPM ID: GPM32310000046) has been previously detected in the proteomic analysis of human CSF (Chiasserini et al., 2014). This motif is highly conserved among different vertebrate species (Supplementary Figure 1). PDPK1 is a master serine/threonine kinase that phosphorylates AGC family of protein kinases (cAMP-dependent, cGMP-dependent and protein kinase C) (Alessi et al., 1997; Chou et al., 1998; Manning and Cantley, 2007). Pelkmans et al. used a high throughput RNA interference combined with infectious virus (vesicular stomatitis virus, VSV) entry and florescent-TF uptake assay to study the human kinases associated with clathrin- and caveolae/raft-mediated endocytosis (Pelkmans et al., 2005). Interestingly, they found silencing CaMKK2 isoform-1 in HeLa cells lead to decreased accumulation of fluorescent-TF in enlarged cytoplasmic structures (data accessible at www.genomernai.org) (Pelkmans et al., 2005). In the same study, silencing PDPK1 resulted in a comparatively lower VSV infection and exhibited toxic effect to TF uptake (Pelkmans et al., 2005). This indicates possible link between PDPK1 and CaMKK2 in regulating TF uptake or trafficking. The function of phosphorylated TF needs future studies to understand any biological relevance.

CaMKK2 is widely expressed in the neurons of rodent CNS and PNS (Ohmstede et al., 1989; Anderson et al., 1998; Sakagami et al., 1998; Vinet et al., 2003). Alternative splicing of exons 14 and/or 16 and usage of differential polyadenylation sites generates several transcriptional isoforms of CaMKK2 that are expressed in a tissue-specific manner (Hsu et al., 2001). The CaMKK2 (+16) transcript is highly enriched in the cerebellum and hippocampus (Ohmstede et al., 1989). Overexpression of (+16)-transcript leads to neurite branching whereas (Δ16)-transcript promoted neurite elongation (Cao et al., 2011). Emerging evidence suggests that altered neurogenesis in the adult hippocampus represents an early event in the course of AD (Mu and Gage, 2011). The Δ14/16 CaMKK2 isoforms lead to loss of detectable kinase activity toward CaMKI and CaMKIV, whereas full-length CaMKK2 isoforms exhibited kinase activity to both effectors as well as autophosphorylation activity (Hsu et al., 2001). CaMKK2 has been implicated in cAMP-responsive element binding protein (CREB) activation and memory consolidation process in the hippocampus (Soderling, 1999; Corcoran and Means, 2001; Soderling and Stull, 2001; Wayman et al., 2008). Loss of CaMKK2 affected the formation of hippocampus-dependent long-term memory (Peters et al., 2003). Therefore, altered CaMKK2 in 3xTg-AD mice cortex may suggest dysfunction of the protein which may affect neurite branching and memory consolidation process.

**Figure 11:**
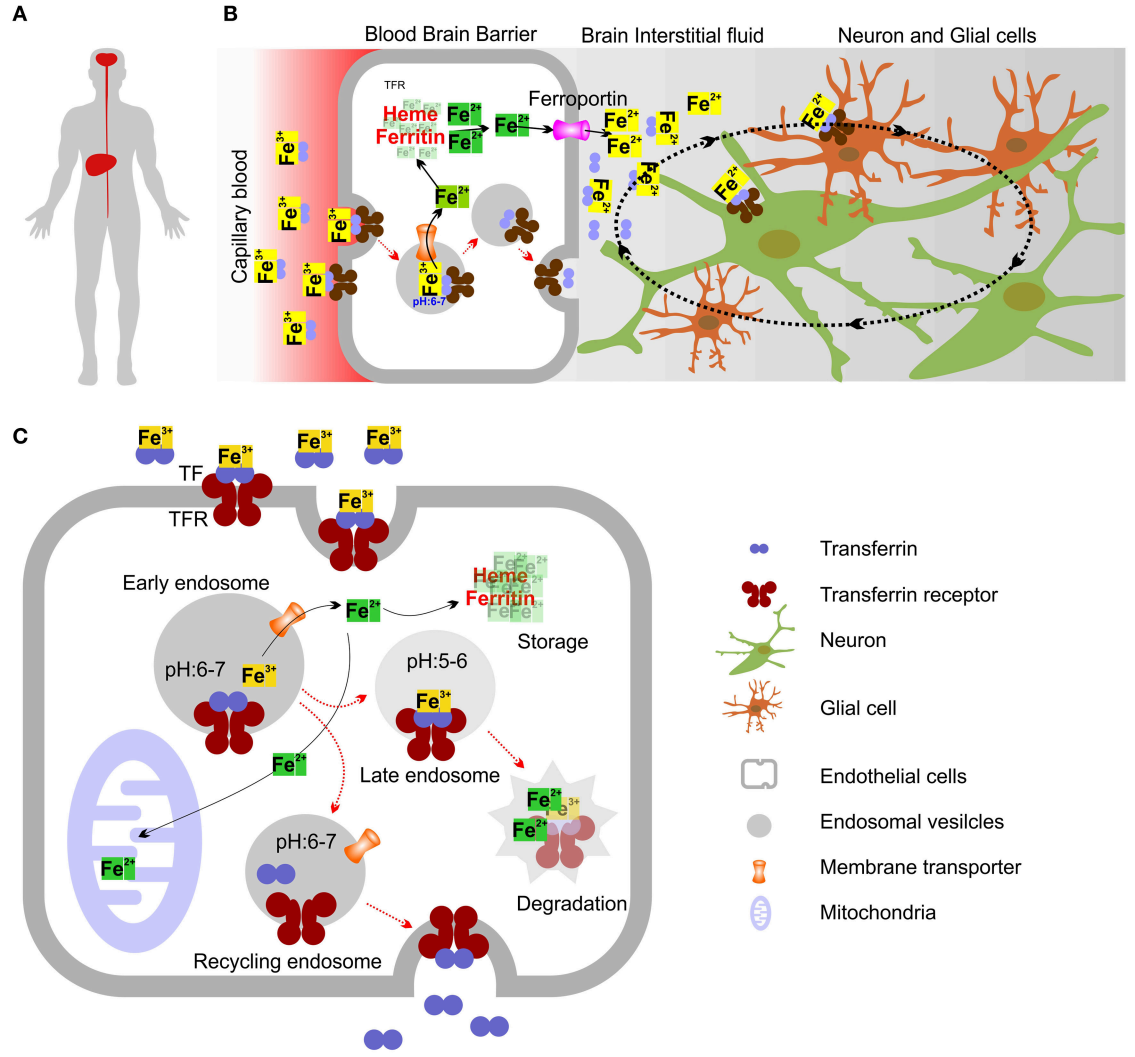
Model showing intracellular iron transport and transport across Blood-Brain Barrier (BBB). **(A)** Diagrammatic representation showing Liver and brain as 2 organs in human involved in the production of TF. **(B)** Mechanism showing how iron crosses the BBB. Holo-TF circulates through the brain capillaries and comes in contact with the luminal TFR which then internalize TF bound iron. The iron exporter ferroportin may allow iron to cross the abluminal membrane of the endothelial cell to enter the interstitial fluid. Most TF in the brain interstitial fluid is synthesized and secreted by oligodendrocytes as Apo-TF. Neurons and glia may acquire most iron through the TFR and holo-TF that is present in the interstitial fluid and cerebrospinal fluid. **(C)** Molecular mechanism showing intracellular iron transport. Most cellular uptake of ferric iron (Fe^3+^) occurs via receptor-mediated endocytosis of TF. Fe^3+^ ions form complexes with the high-affinity iron binding TF, which then binds to TFR. After endocytosis of TFR, the acidic environment of the early endosomes triggers the release of Fe^3+^ from the TF-TFR complex, which is recycled to the plasma membrane via recycling endosomes. Ferric reductases localized to the endosome reduce Fe^3+^ (ferric) to its Fe^2+^ (ferrous) form before Fe^2+^ is released into the cytosol by the divalent metal transporter-1 (DMT1) in an H^+^-dependent manner. Fe^3+^ may also be sorted into late endosomes (LE) and lysosomes (LY), where it is reduced to Fe^2+^ by ferric reductases. In LE and LY, Fe^2+^ can also be released by other endo-lysosomal iron release channels/transporters. Iron can then bind to the chaperones that donate iron to specific target proteins (not shown) (Philpott, 2012) or enter mitochondria through the dedicated mitochondrial iron transporters (Chen et al., 2009), where it is used for the synthesis of iron–sulfur clusters and haem (Rouault, 2013). Iron can also be stored in cytosolic proteins such as ferritin, which can sequester up to 4,500 iron atoms (Arosio et al., 2009). Ferritin sequestration of iron prevents free iron from reaching high concentrations in the cytosolic and nuclear compartments (Arosio et al., 2009). Degradation of ferritin in lysosomes leads to the formation of disorganized iron-rich deposits known as haemosiderin (not shown) (Cohen et al., 2010).

The CaMKK2 and TF expression pattern are overlapped in the human nervous system and liver tissues (Supplementary Figures 5, 6). This indicates that dysfunctional CaMKK2 may directly impact TF abundance and phosphorylation which in turn may alter brain iron level leading to neurodegeneration. Cells in the nervous system do not have direct access to nutrients, including iron (Rouault, 2013). TF secreted by the liver in the serum function to deliver iron to different organs including brain, see a model in Figures 11A,B (Sawaya and Schaeffer, 1995). TF and TFR mediate uptake of iron across the blood-brain-barrier (BBB) (Brightman et al., 1970; Ballabh et al., 2004). The generally agreed mechanism is that the brain capillary endothelial cells absorb iron-bound TF (holo-TF) from the blood via TFR mediated endocytosis(Bradbury, 1997) (Figures 11B,C). After endocytosis, the acidic environment of the early endosomes triggers the release of Fe^3+^ from the TF-TFR complex, which is recycled to the plasma membrane via recycling endosomes (Mills et al., 2010). How iron subsequently exits the brain capillary endothelial cells and reach the brain extracellular fluid is less well understood (Altamura and Muckenthaler, 2009). Interestingly, CaMKK2 has been known to confer protection to the endothelial cells that primarily constitute BBB (Marcelo et al., 2016). CaMKK2 KO mice displayed loss of BBB collagen IV protein and reduced expression of extracellular matrix-degrading, Ca^2+^dependent matrix metalloproteinases (MMP) 2/9 (Mccullough et al., 2013; Liu et al., 2014). Therefore, it is possible that aberrant CaMKK2 may alter the TF cycle through the BBB.

Histological analysis revealed that aberrant extracellular TF is distributed around the senile plaques in human AD brain tissues which stained positive for iron accumulation (Connor et al., 1992). High iron concentration in the brain and mutations in the genes associated with iron metabolism were found associated with neurodegenerative disorders (AD, PD, and HD) which suggest that iron misregulation in the brain plays a part in the neuronal death (Ke and Ming Qian, 2003). Neurodegeneration with brain iron accumulation (NBIA) is a group of neurodegenerative diseases characterized by most severe iron accumulation in the cerebellum and cerebellar atrophy (reduction in Purkinje cells) in some subtypes (Gregory and Hayflick, 1993; Levi and Finazzi, 2014; Arber et al., 2016). CaMKK2 KO mice exhibited morphological and physiological deficits in the Purkinje cells (Ribar et al., 2000; Kokubo et al., 2009). Dysregulation of the intracellular Ca^2+^ homeostasis was suggested underlying the development of AD (Thibault et al., 2007; Hermes et al., 2010; Berridge, 2011). The two pathological hallmarks of AD are the presence of neurofibrillary tangles (NFT) made of aggregates of the hyperphosphorylated tau protein and of amyloid plaques composed of Aβ peptides, primarily Aβ1-40 and Aβ1-42 (Ballatore et al., 2007; Serrano-Pozo et al., 2011; Doig et al., 2017). The failure of all drugs targeting Aβ lead to the necessity of alternative hypothesis (Doig et al., 2017). The calcium hypothesis of AD has now become more relevant in the recent years (Alzheimer's Association Calcium Hypothesis, 2017). The molecular mechanisms that regulate temporal and spatial neuronal Ca^2+^, the role of altered Ca^2+^ in various subcellular compartments and how such alterations affect the performance of neurons under various conditions, ranging from healthy state to deterioration in performance during aging or AD has become a major focus of research (Alzheimer's Association Calcium Hypothesis, 2017). This study clearly showed that loss of CaMKK2 leads to cholinergic signaling mediated abnormal Ca^2+^ response which may be associated with abnormal TF trafficking (Supplementary Figure 2). However, the exact causative factor for Ca^2+^ dysregulation during the progression of AD is not known. It has been shown that Aβ peptides (Aβ 1-42) alter intracellular Ca^2+^ through NMDA receptors in mouse cortical neurons which in turn lead to the phosphorylation of tau by AMPK through CaMKK2-AMPK signaling pathway (Thornton et al., 2011; Mairet-Coello et al., 2013). In the present study, the amount of TF was found significantly increased in the olfactory bulb and cerebellum of CaMKK2 KO mice (Figure 5). This provides a link to the olfaction (Esiri and Wilcock, 1984; Kovacs et al., 2001; Zou et al., 2016), voluntary motor activity and motor learning (Guo et al., 2016; Jacobs et al., 2018) pathologically affected during early stages of AD. A significant decrease of TF in the CaMKK2 KO liver indicates that liver may play role in the development of AD. Presenilin 2 (Psen2) is a component of the γ-secretase activity responsible for generating Aβ by proteolysis. It has been shown that Psen2 mRNA accumulation takes place in the liver but not in the brain, suggesting liver as the origin of brain Aβ (Sutcliffe et al., 2011). Taking together all these findings, it can be suggested that dysregulation of CaMKK2 in the brain and liver may lead to aberrant TF trafficking and turnover which in turn imbalance the iron homeostasis in the brain and lead to the disease.

AD is one of the most prevalent dementia worldwide (Sharma and Singh, 2016). The current diagnostic criteria for AD has included CSF biomarkers (Aβ peptides, Tau and P-Tau) which are obtained by invasive lumbar punctures that cause nausea, severe backache, and weakness in elderly people (Lehmann and Teunissen, 2016). There is an urgent need for minimal invasive serum-based biomarker that has a significant advantage in time- and cost-efficiency as well as patient acceptance (Sharma and Singh, 2016; O'bryant et al., 2017). Plasma proteins of amyloid pathology, circulatory miRNAs, cytokines, kinases, axonal proteins, lipids, and fragments of already known AD markers are currently investigated for their potential as blood-based AD biomarker (Lista et al., 2013; Huynh and Mohan, 2017). In addition, serum TF level (Squitti et al., 2010), desaturation level of serum TF-iron (Hare et al., 2015), glycosylated-TF in CSF (Guevara et al., 1998; Van Rensburg et al., 2000; Taniguchi et al., 2008; Shirotani et al., 2011) and serum (Yu et al., 2003) has been proposed as potential AD biomarkers. The present study has shown that the total TF level in the CSF of postmortem AD patients decreased compared to age-matched control but the serum TF level remained unaltered which makes total TF level unsuitable as a diagnostic biomarker. In addition, loss of CaMKK2 mediated altered TF HMW fractions is an indication that glycosylated-TF (HMW) may reflect the diseased state in AD. However, use of glycosylated-TF as a diagnostic biomarker is a daunting analytical challenge due to inherent complexity and variability in the glycans which is highlighted in this study (Zhang et al., 2016). The consistent reduction of P-TF (pH~3-4 fraction) in the serum of CaMKK2 KO mice, 3xTg-AD mice, and in the CSF and serum from EOAD and LOAD postmortem human patients indicate that P-TF (pH~3-4 fraction) profile is a promising novel biomarker for AD.

Overall, this study indicates a novel link between CaMKK2 mediated regulation of TF trafficking and tissue-specific turnover which provides a mechanistic insight into the neurodegeneration process. Loss of CaMKK2 leads to a decrease in the pH~3-4 fraction of TF containing multiple phosphorylated residues and overall data indicate that CaMKK2 is not the kinase directly involved in TF phosphorylation. In addition, this study also indicates that aberrant CaMKK2 due to potentially dysregulated Ca^2+^ homeostasis in the AD may lead to decreased secretion of phosphorylated TF (pH~3-4) in the CSF and serum which can be used as a biomarker.

## Ethics statement

This study was carried out in accordance with the recommendations of NIH Neurobiobank with written informed consent from all subjects. All subjects gave written informed consent in accordance with the Declaration of Helsinki. The protocol was approved by the St. Boniface Hospital Albrechtsen Research Centre Biosafety committee.

## Author contributions

MS conceptualized, designed and performed the experiments, analyzed the data and wrote the manuscript.

### Conflict of interest statement

MS filed a US provisional patent on 4th October 2018 (Serial number: 62/741,148) entitled “Novel biomarker for Alzheimer's disease in human.”

## Supplementary material

Supplementary Figure 1 Conservation of TF amino acid sequence in vertebrate species and mapping of the P-TF residues identified by mass spectrometry. **(A)** Clustal Omega Sequence alignment (Mcwilliam et al., 2013) showing alignment of mammalian TF protein sequences (Swiss-Prot manually annotated and reviewed sequences only: http://www.uniprot.org) from Homo sapiens (Human), Pan troglodytes (Chimpanzee), Mus musculus (Mouse), Rattus norvegicus (Rat), Sus scrofa (Pig), Oryctolagus cuniculus (Rabbit), Bos taurus (Bovine) and Equus caballus (Horse). Left column indicates UniProt unique protein identifier number. Asterisk indicates positions which have a single, fully conserved residue. Colon indicates conservation between groups of strongly similar properties. Period indicates conservation between groups of weakly similar properties. Color highlights showing residues in N and C subdomain of human TF identified as interacting with hTFR. Specific residues identified by cryo-EM (yellow rectangle) (Cheng et al., 2004) and epitope mapping (pink rectangle) (Teh et al., 2005) may interact with TFR. Orange: overlap between cryo-EM (yellow) and epitope mapping (pink), green highlighted residues in the N-lobe that may interact with the human TFR(Cheng et al., 2004). Each of the homologous N and C terminal domains of TF binds one ion of ferric iron. The amino acid residues involved in TF iron binding are mentioned by light pink highlights (Wally et al., 2006; Noinaj et al., 2012; Wang et al., 2015). **(B)** Phylogenetic tree of TF. The horizontal lines are branches and represent evolutionary lineages changing over time. The values indicate the length of the branch that represents an amount genetic change equivalent to the length. The units of the branch length are usually nucleotide substitutions per site (the number of changes or “substitutions” divided by the length of the sequence). The phylogenetic tree was constructed using Clustal Omega web tool.

Supplementary Figure 2 Muscarine treatment induced intracellular calcium response in wild-type and CaMKK2 KO HEK293 cells. **(A,D)** Line graphs showing time-lapse Fluo-4 intensities in wild-type and CaMKK2 KO HEK293 cells. Each thin line indicates data obtained using from a single cell. The thick line represents the mean. *N* = 12. Detailed methodology is mentioned in the text. **(B,C,E,F)** Scatter plot showing Fluo-4 intensities in wild-type and CaMKK2 KO HEK293 cells following 3.5 and 10 min of 10 μM muscarine. Data represent Mean ± SEM. The *p*-value by *t*-test (unpaired).

Supplementary Figure 3 Relative abundance of total TF in the CSF and serum samples obtained from postmortem human EOAD and LOAD patients and age matched unaffected controls. **(A,C,G,H)** Immunoblots showing expression of total TF the CSF and serum samples. Bottom panels represent SDS-PAGE gel stained with Oriole to show total protein loading. Black arrow indicates the band used for normalization of TF expression. **(B)** Scatter plot showing relative abundance of the TF in the CSF based on immunoblots shown in **(A)**. **(D,F)** Scatter plot showing relative abundance of the TF in the CSF and matched serum from the same individuals based on the immunoblots shown in **(C)**. Black arrows in stained gel indicates the band used for normalization of TF expression. **(G,H)** Immunoblots showing expression of total TF the CSF and serum samples. Bottom panels represent SDS-PAGE gel stained with Oriole to show total protein loading.

Supplementary Figure 4 TF model showing surface accessibility of identified P-TF residues and overlapping with kinase motifs. **(A)** Molecular surface representation of the overall structure of Fe_N_Fe_C_-hTF with subdomains N1 in blue, N2 in green, C1 in yellow, C2 in red, and peptide linker in brown (based on PDB:3QYT). Molecular graphics were prepared using UCSF Chimera package (Pettersen et al., 2004). **(B)** Ribbon and molecular surface representation of hTF showing TFR binding site (Wally et al., 2006) (pink) and potential PDK1 binding motif (orange). **(C)** Molecular surface presentations of hTF showing TFR binding site and potential PDK1 binding motif as accessible. **(D)** List of protein motifs predicted for TF (SwissProt entry P02787) using the Scansite cell signaling interactions prediction “MotifScan” module (https://myhits.isb-sib.ch/cgi-bin/motif_scan) using medium stringency (Obenauer et al., 2003).

Supplementary Figure 5 Human transcriptome and proteome data mining revealed TF and CaMKK2 has overlapping expression pattern in human brain and liver tissues. **(A,B)** Whisker plot showing CaMKK2 and TF RNA expression in different tissues. The expression values are shown in TPM (transcripts per million) calculated from a gene model with isoforms collapsed to a single gene. **(C)** Bar graph showing the number of RNA sequencing samples for different tissues used in generating the datasets presented in **(A,B)**. The data was obtained from the Genotype-Tissue Expression Project (GTEx Portal) on Dec/14/2017. RNA sequencing of coding RNAs data accessed from Genotype-Tissue Expression (GTEx) Project data portal (https://gtexportal.org/home/) (Fagerberg et al., 2014).

Supplementary Figure 6 Human Protein Atlas data mining showing expression of CaMKK2 and TF in human brain and liver tissues by immunohistochemistry. **(A,B)** Immunostained section of human cerebral cortex and liver tissues showing strong CaMKK2 expression in neuronal cells and medium CaMKK2 expression was noted in hepatocytes. Polyclonal affinity isolated rabbit anti-CaMKK2 and anti-TF antibodies (catalog number: HPA017389 and HPA001527, respectively, Sigma) were used for immunodetection. Image credit: Human Protein Atlas. Images are available at Human Protein Atlas website version 18 (www.proteinatlas.org).
