# Supplementary figures and images for: Loss of Ca2+/Calmodulin Dependent Protein Kinase Kinase 2 Leads to Aberrant Transferrin Phosphorylation and Trafficking: A Potential Biomarker for Alzheimer's Disease
Source: Front Mol Biosci. 2018 Nov 20;5:99. doi: 10.3389/fmolb.2018.00099 (PMC6256988; doi:10.3389/fmolb.2018.00099)

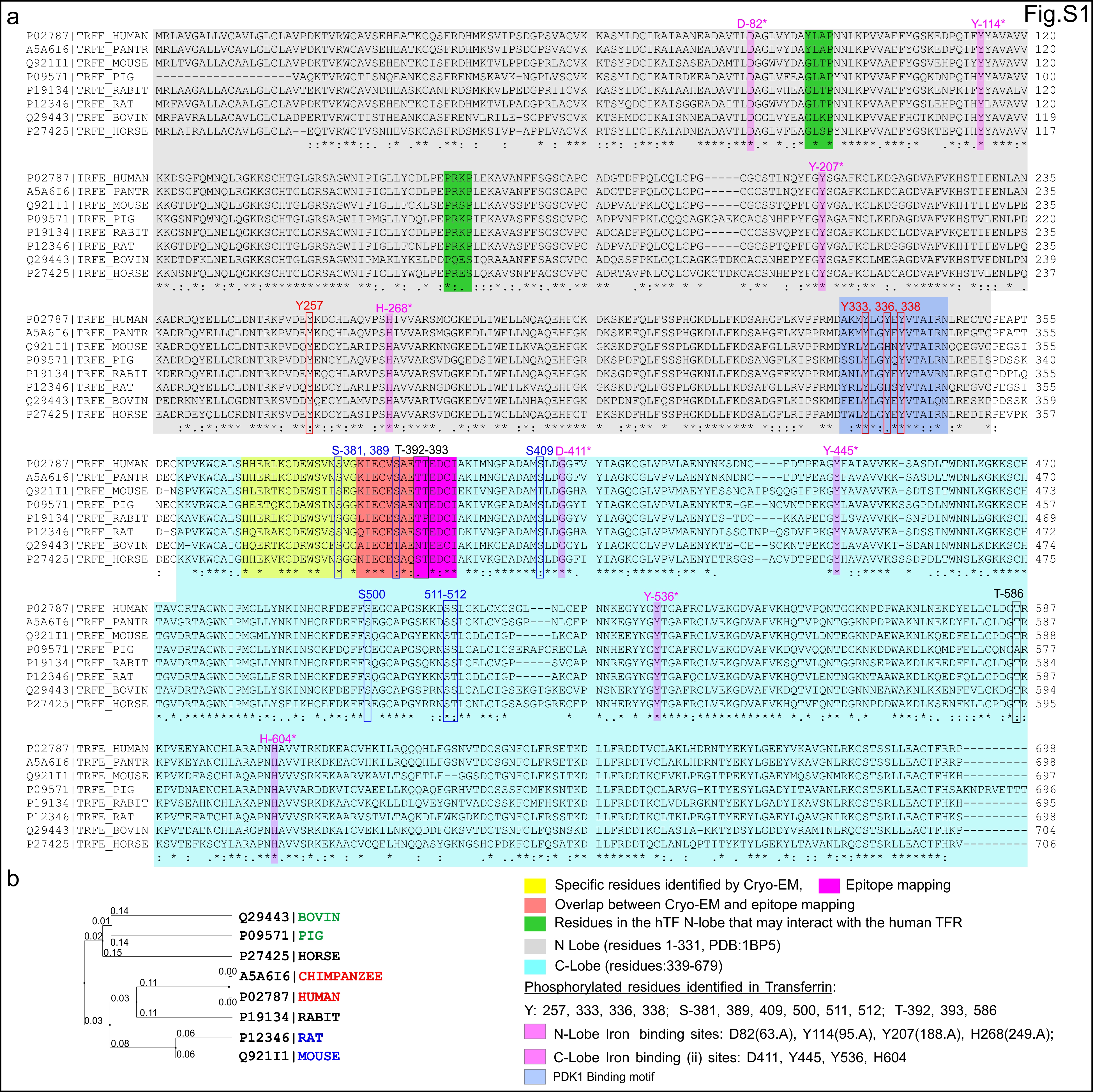

Supplement: Supplementary Figure 1 — Conservation of TF amino acid sequence in vertebrate species and mapping of the P-TF residues identified by mass spectrometry. (A) Clustal Omega Sequence alignment (Mcwilliam et al., 2013) showing alignment of mammalian TF protein sequences (Swiss-Prot manually annotated and reviewed sequences only: http://www.uniprot.org) from Homo sapiens (Human), Pan troglodytes (Chimpanzee), Mus musculus (Mouse), Rattus norvegicus (Rat), Sus scrofa (Pig), Oryctolagus cuniculus (Rabbit), Bos taurus (Bovine) and Equus caballus (Horse). Left column indicates UniProt unique protein identifier number. Asterisk indicates positions which have a single, fully conserved residue. Colon indicates conservation between groups of strongly similar properties. Period indicates conservation between groups of weakly similar properties. Color highlights showing residues in N and C subdomain of human TF identified as interacting with hTFR. Specific residues identified by cryo-EM (yellow rectangle) (Cheng et al., 2004) and epitope mapping (pink rectangle) (Teh et al., 2005) may interact with TFR. Orange: overlap between cryo-EM (yellow) and epitope mapping (pink), green highlighted residues in the N-lobe that may interact with the human TFR(Cheng et al., 2004). Each of the homologous N and C terminal domains of TF binds one ion of ferric iron. The amino acid residues involved in TF iron binding are mentioned by light pink highlights (Wally et al., 2006; Noinaj et al., 2012; Wang et al., 2015). (B) Phylogenetic tree of TF. The horizontal lines are branches and represent evolutionary lineages changing over time. The values indicate the length of the branch that represents an amount genetic change equivalent to the length. The units of the branch length are usually nucleotide substitutions per site (the number of changes or “substitutions” divided by the length of the sequence). The phylogenetic tree was constructed using Clustal Omega web tool. [file Image_1.JPEG]

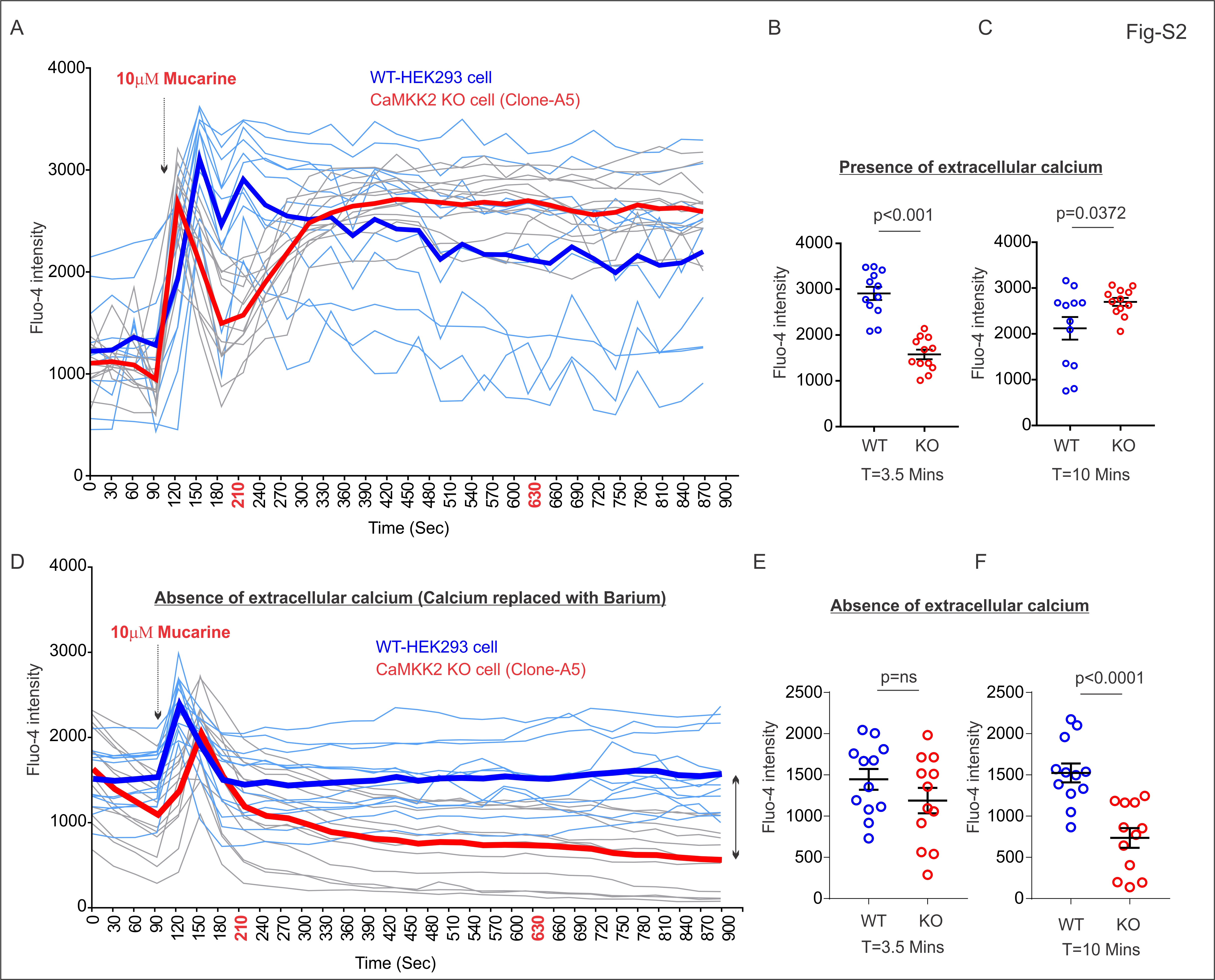

Supplement: Supplementary Figure 2 — Muscarine treatment induced intracellular calcium response in wild-type and CaMKK2 KO HEK293 cells. (A,D) Line graphs showing time-lapse Fluo-4 intensities in wild-type and CaMKK2 KO HEK293 cells. Each thin line indicates data obtained using from a single cell. The thick line represents the mean. N = 12. Detailed methodology is mentioned in the text. (B,C,E,F) Scatter plot showing Fluo-4 intensities in wild-type and CaMKK2 KO HEK293 cells following 3.5 and 10 min of 10 μM muscarine. Data represent Mean ± SEM. The p-value by t-test (unpaired). [file Image_2.JPEG]

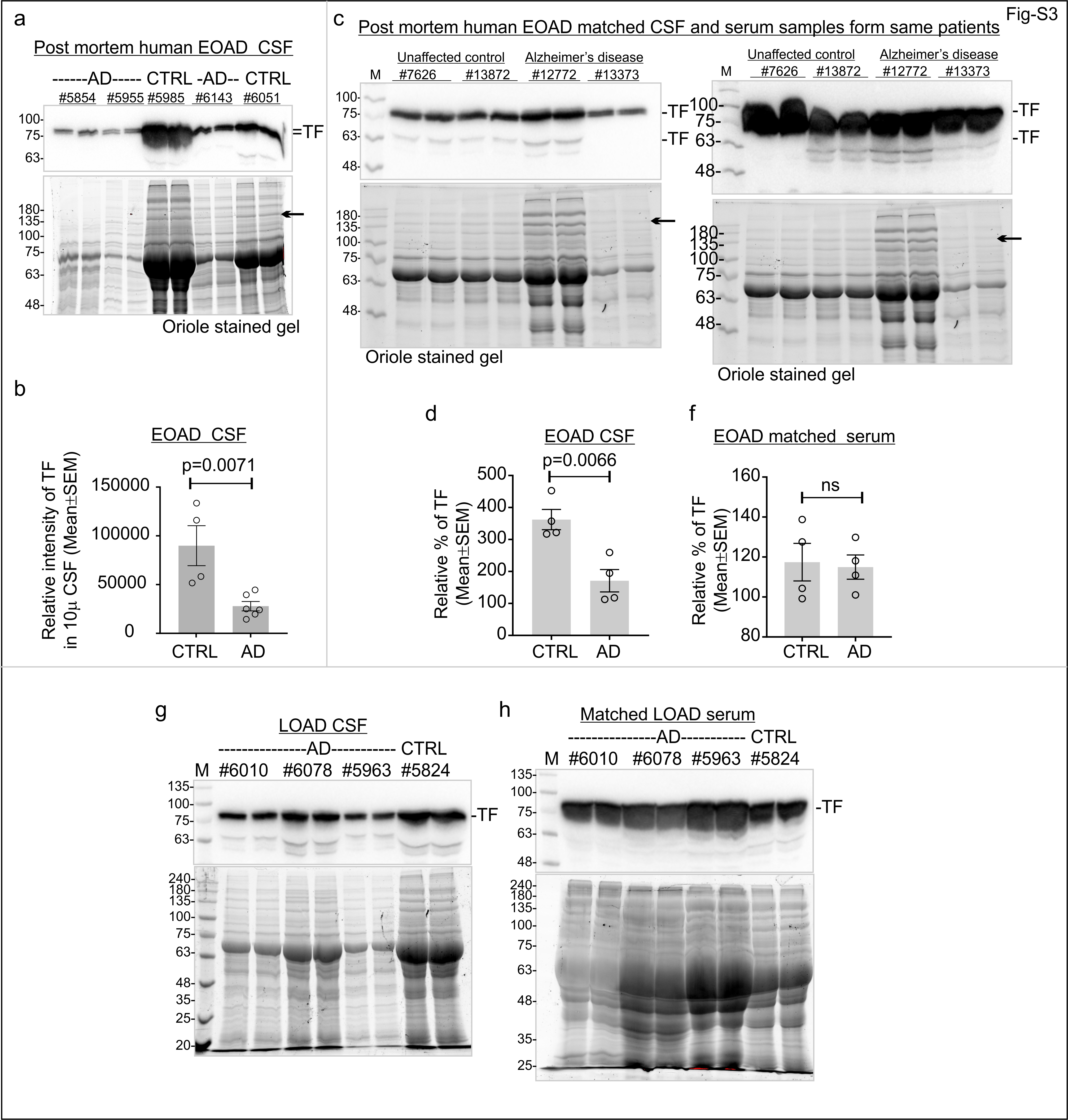

Supplement: Supplementary Figure 3 — Relative abundance of total TF in the CSF and serum samples obtained from postmortem human EOAD and LOAD patients and age matched unaffected controls. (A,C,G,H) Immunoblots showing expression of total TF the CSF and serum samples. Bottom panels represent SDS-PAGE gel stained with Oriole to show total protein loading. Black arrow indicates the band used for normalization of TF expression. (B) Scatter plot showing relative abundance of the TF in the CSF based on immunoblots shown in (A). (D,F) Scatter plot showing relative abundance of the TF in the CSF and matched serum from the same individuals based on the immunoblots shown in (C). Black arrows in stained gel indicates the band used for normalization of TF expression. (G,H) Immunoblots showing expression of total TF the CSF and serum samples. Bottom panels represent SDS-PAGE gel stained with Oriole to show total protein loading. [file Image_3.JPEG]

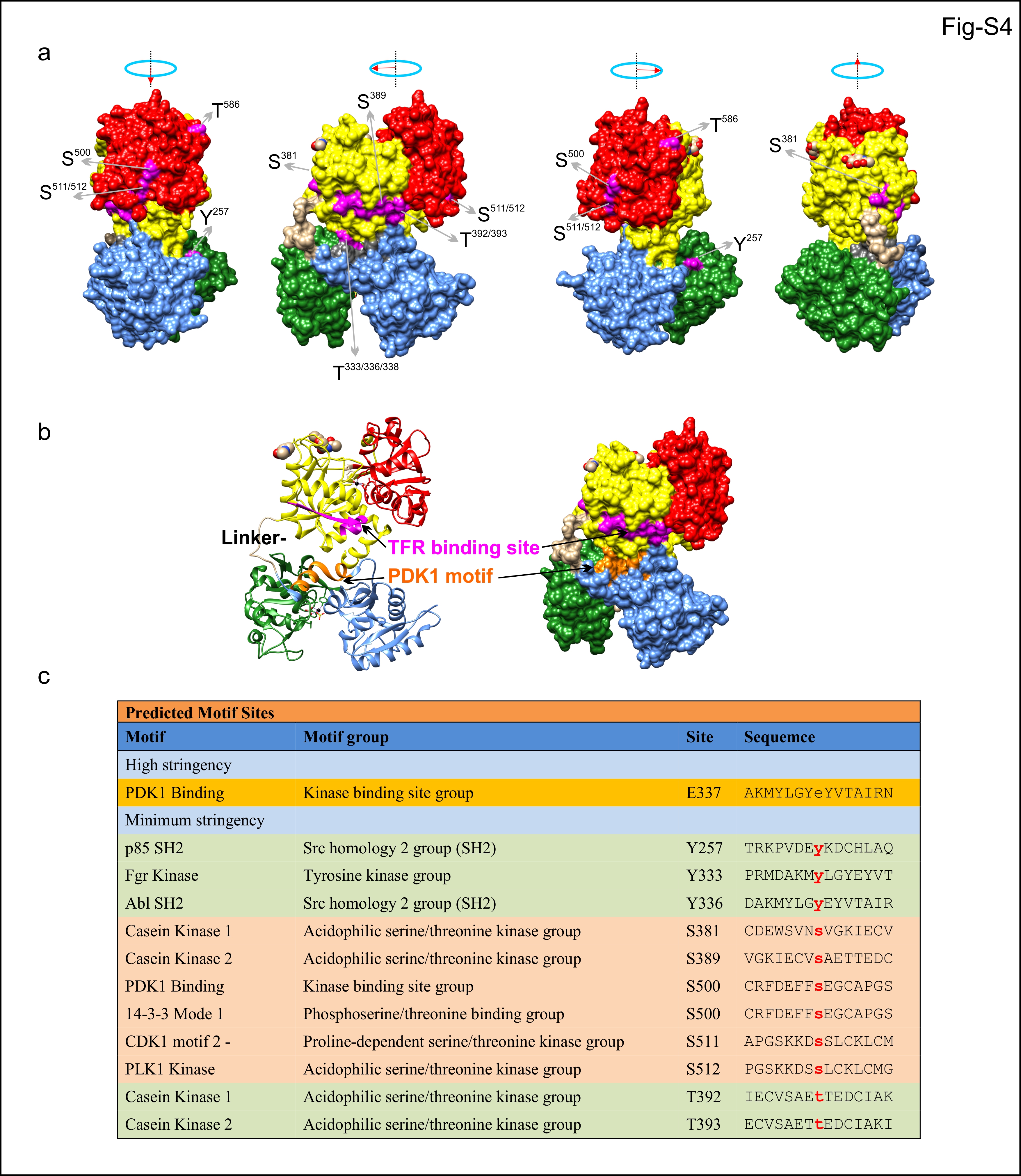

Supplement: Supplementary Figure 4 — TF model showing surface accessibility of identified P-TF residues and overlapping with kinase motifs. (A) Molecular surface representation of the overall structure of FeNFeC-hTF with subdomains N1 in blue, N2 in green, C1 in yellow, C2 in red, and peptide linker in brown (based on PDB:3QYT). Molecular graphics were prepared using UCSF Chimera package (Pettersen et al., 2004). (B) Ribbon and molecular surface representation of hTF showing TFR binding site (Wally et al., 2006) (pink) and potential PDK1 binding motif (orange). (C) Molecular surface presentations of hTF showing TFR binding site and potential PDK1 binding motif as accessible. (D) List of protein motifs predicted for TF (SwissProt entry P02787) using the Scansite cell signaling interactions prediction “MotifScan” module (https://myhits.isb-sib.ch/cgi-bin/motif_scan) using medium stringency (Obenauer et al., 2003). [file Image_4.JPEG]

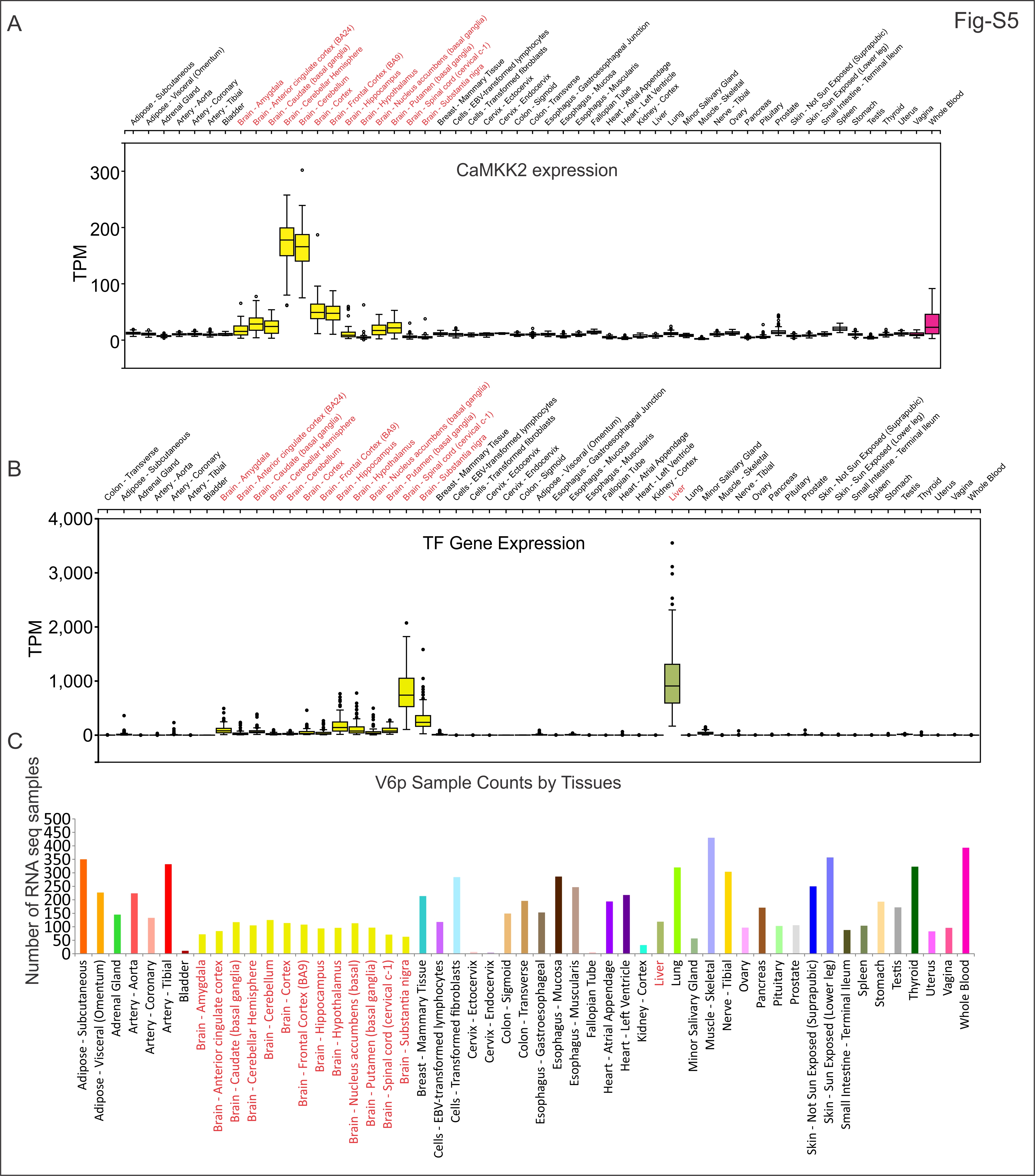

Supplement: Supplementary Figure 5 — Human transcriptome and proteome data mining revealed TF and CaMKK2 has overlapping expression pattern in human brain and liver tissues. (A,B) Whisker plot showing CaMKK2 and TF RNA expression in different tissues. The expression values are shown in TPM (transcripts per million) calculated from a gene model with isoforms collapsed to a single gene. (C) Bar graph showing the number of RNA sequencing samples for different tissues used in generating the datasets presented in (A,B). The data was obtained from the Genotype-Tissue Expression Project (GTEx Portal) on Dec/14/2017. RNA sequencing of coding RNAs data accessed from Genotype-Tissue Expression (GTEx) Project data portal (https://gtexportal.org/home/) (Fagerberg et al., 2014). [file Image_5.JPEG]

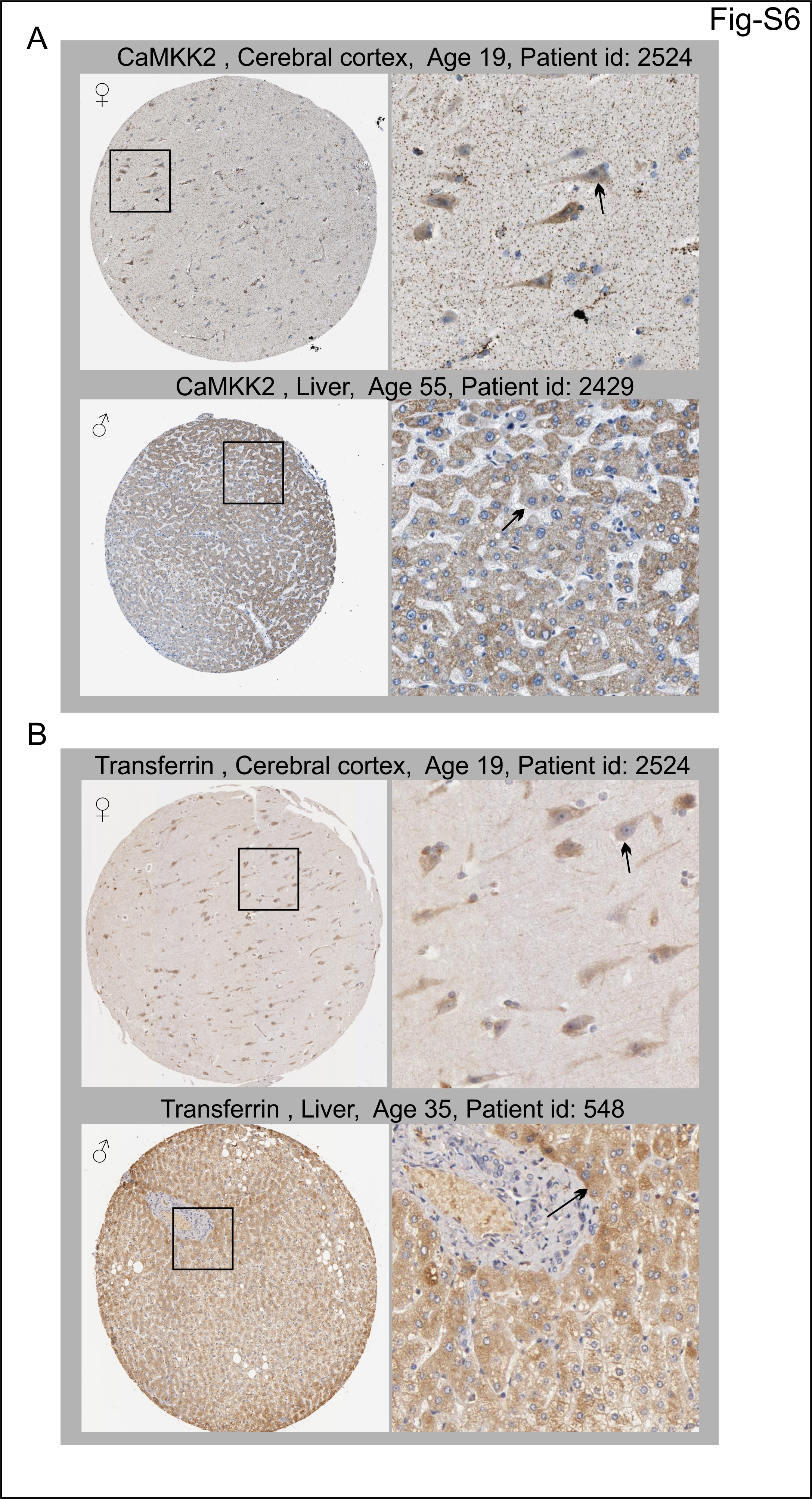

Supplement: Supplementary Figure 6 — Human Protein Atlas data mining showing expression of CaMKK2 and TF in human brain and liver tissues by immunohistochemistry. (A,B) Immunostained section of human cerebral cortex and liver tissues showing strong CaMKK2 expression in neuronal cells and medium CaMKK2 expression was noted in hepatocytes. Polyclonal affinity isolated rabbit anti-CaMKK2 and anti-TF antibodies (catalog number: HPA017389 and HPA001527, respectively, Sigma) were used for immunodetection. Image credit: Human Protein Atlas. Images are available at Human Protein Atlas website version 18 (www.proteinatlas.org). [file Image_6.JPEG]
